# Bone protein “extractomics”: comparing the efficiency of bone protein extractions of *Gallus gallus* in tandem mass spectrometry, with an eye towards paleoproteomics

**DOI:** 10.7717/peerj.2603

**Published:** 2016-10-27

**Authors:** Elena R. Schroeter, Caroline J. DeHart, Mary H. Schweitzer, Paul M. Thomas, Neil L. Kelleher

**Affiliations:** 1 Department of Biological Sciences, North Carolina State University, Raleigh, NC, United States; 2 Proteomics Center of Excellence and Departments of Chemistry, Molecular Biosciences, and the Feinberg School of Medicine, Northwestern University, Evanston, IL, United States

**Keywords:** Bone matrix proteins, Bone protein extraction protocols, Methods comparison, Paleoproteomics, Mass spectrometry, Proteomics

## Abstract

Proteomic studies of bone require specialized extraction protocols to demineralize and solubilize proteins from within the bone matrix. Although various protocols exist for bone protein recovery, little is known about how discrete steps in each protocol affect the subset of the bone proteome recovered by mass spectrometry (MS) analyses. Characterizing these different “extractomes” will provide critical data for development of novel and more efficient protein extraction methodologies for fossils. Here, we analyze 22 unique sub-extractions of chicken bone and directly compare individual extraction components for their total protein yield and diversity and coverage of bone proteins identified by MS. We extracted proteins using different combinations and ratios of demineralizing reagents, protein-solubilizing reagents, and post-extraction buffer removal methods, then evaluated tryptic digests from 20 µg aliquots of each fraction by tandem MS/MS on a 12T FT-ICR mass spectrometer. We compared total numbers of peptide spectral matches, peptides, and proteins identified from each fraction, the redundancy of protein identifications between discrete steps of extraction methods, and the sequence coverage obtained for select, abundant proteins. Although both alpha chains of collagen I (the most abundant protein in bone) were found in all fractions, other collagenous and non-collagenous proteins (e.g., apolipoprotein, osteonectin, hemoglobin) were differentially identified. We found that when a standardized amount of extracted proteins was analyzed, extraction steps that yielded the most protein (by weight) from bone were often *not* the ones that produced the greatest diversity of bone proteins, or the highest degree of protein coverage. Generally, the highest degrees of diversity and coverage were obtained from demineralization fractions, and the proteins found in the subsequent solubilization fractions were highly redundant with those in the previous fraction. Based on these data, we identify future directions and parameters to consider (e.g., proteins targeted, amount of sample required) when applying discrete parts of these protocols to fossils.

## Introduction

Molecular sequence data provide critical information for studies of evolutionary history, phylogenetic relationships, disease states, and other aspects of the biology of extant organisms. Additionally, molecular sequences recovered from fossils have the potential to shed light on the origin of evolutionary novelties (e.g., feathers), resolve evolutionary relationships (e.g., Buckley, 2015; Welker et al., 2015), and identify indeterminate or fragmentary fossil elements to the taxon that produced them (Buckley et al., 2010). However, until recently, molecular data were thought to be inaccessible from fossil remains, particularly those older than one million years (Schweitzer, Schroeter & Goshe, 2014 and references therein). Proteomic analyses of archeological and paleontological bone tissues are fraught with many challenges that hinder the widespread application of these techniques to fossils. Primary among these challenges is the inherent uncertainty surrounding the nature of the fossils themselves, which have undergone processes that are impossible to directly measure or observe, but which profoundly affect the chemistry of organic matter remaining within them. These include, but are not limited to, uncertainty in the rates at which proteins may degrade over geologic time, and the role that any given environmental factor plays in protein preservation and/or degradation (Hedges, 2002; Nielsen-Marsh et al., 2000; Nielsen-Marsh & Hedges, 2000), uncertainty regarding diagenetic modifications that might alter the chemical structure of ancient molecules (e.g., AGEs) (Cleland, Schroeter & Schweitzer, 2015; Nielsen-Marsh et al., 2005; Van Klinken & Hedges, 1995), and finally, uncertainty over what fluctuations in the biological, thermal, and hydrological conditions a fossil may have been subjected to over thousands to millions of years, and how these may affect their molecular composition (Schroeter & Cleland, 2016; Smith et al., 2003). However, although molecular studies on fossil remains will always be laden with unknown (and unknowable) variables that must be studied indirectly, the nascent field of molecular paleontology is also affected by unexplored gaps in our knowledge which *must* be addressed and resolved for the field to continue to progress.

One such gap is the incomplete understanding of how different protein extraction protocols ultimately affect the results produced in proteomic studies of fossil bone. Bone is a composite tissue comprising an organic matrix (approximately 90% collagen I and 10% other, non-collagenous proteins (NCPs) biomineralized with hydroxyapatite (Ca_5_(PO_4_)_3_OH) (Weiner & Wagner, 1998; Young, 2003; Zylberberg, 2004). Biomineralization confers stability and stiffness to bone tissue (Gautieri et al., 2011; Wang et al., 2012; Weiner & Wagner, 1998), and is hypothesized to confer resistance to enzymatic digestion of the proteinaceous component (Collins et al., 2002; Trueman & Martill, 2002) that may allow it to persist over geologic time (Schweitzer, Schroeter & Goshe, 2014). However, this intimate mineral association also requires the use of specialized extraction protocols to recover proteins from the bone matrix (Cleland, Voegele & Schweitzer, 2012). A variety of reagents have been employed to remove bone mineral and increase access to remaining organic fractions, such as hydrochloric acid (HCl) (Buckley, 2013; Buckley, 2015; Buckley et al., 2009; Buckley et al., 2010; Buckley, Larkin & Collins, 2011; Buckley & Wadsworth, 2014; Cleland, Schroeter & Schweitzer, 2015; Cleland, Voegele & Schweitzer, 2012; Ostrom et al., 2000; Wadsworth & Buckley, 2014; Welker et al., 2015), sodium ethylenediaminetetraacetic acid (EDTA) (Buckley et al., 2008; Cappellini et al., 2012; Cleland, Voegele & Schweitzer, 2012; Humpula et al., 2007; Nielsen-Marsh et al., 2002; Nielsen-Marsh et al., 2005; Ostrom et al., 2006; Schweitzer et al., 2007; Schweitzer et al., 2009), and ammonium EDTA (Ostrom et al., 2000). Reagents used to solubilize bone proteins for subsequent mass spectrometry (MS) and immunological applications are equally diverse, and have included (among others) ammonium bicarbonate (ABC) (Buckley et al., 2009; Buckley et al., 2010; Buckley, Larkin & Collins, 2011; Cappellini et al., 2012; Cleland, Schroeter & Schweitzer, 2015; Cleland, Voegele & Schweitzer, 2012), and guanidine hydrochloride (GuHCl) (Buckley, 2015; Buckley & Wadsworth, 2014; Cleland, Voegele & Schweitzer, 2012; Schweitzer et al., 2007; Schweitzer et al., 2009; Wadsworth & Buckley, 2014). However, these methods have not been consistently employed across fossil studies, which may hamper direct comparisons of preserved protein content between fossil specimens, as these reagents have been shown to vary in efficacy when used to extract bone proteins for enzyme-linked immunosorbent assay (ELISA) and sodium dodecyl sulfate polyacrylamide gel electrophoresis (SDS-PAGE) (Cleland, Voegele & Schweitzer, 2012). One might expect these reagents to produce similar disparities in MS analyses, but little is known about how discrete steps in the respective protocols affect absolute protein yield, or which subset of the bone proteome is preferentially recovered by each method. Indeed, MS analyses of other tissues (e.g., insect tissue (Cilia et al., 2009), mouse cartilage (Wilson & Bateman, 2008)) have shown that different reagents solubilize distinct subsets of the tissue’s proteome (i.e., an “extractome”), suggesting that the choice of extraction reagent alone can greatly affect the regions of the proteome identified by MS. Therefore, the resulting protein identifications may not reflect the entirety of what is actually present in a sample.

Here, we begin to address this last issue through an in-depth comparison of extractomes identified from discrete steps of protein extractions from chicken bone. Little is known about how variations in protocol affect MS results for *fresh* bone, let alone fossil bone. Thus, it is first necessary to test the efficiencies of extraction methodologies on extant tissues to establish any disparity between protocols themselves, absent the chemical influences of diagenesis that can alter a fossil proteome in a highly variable manner between individual fossils. Establishing this baseline provides critical data for future development of novel and more efficient protocols for extant bone and insight into discrete protocol steps that may be promising for further testing for their utility in paleoproteomic investigations.

**Figure 1:**
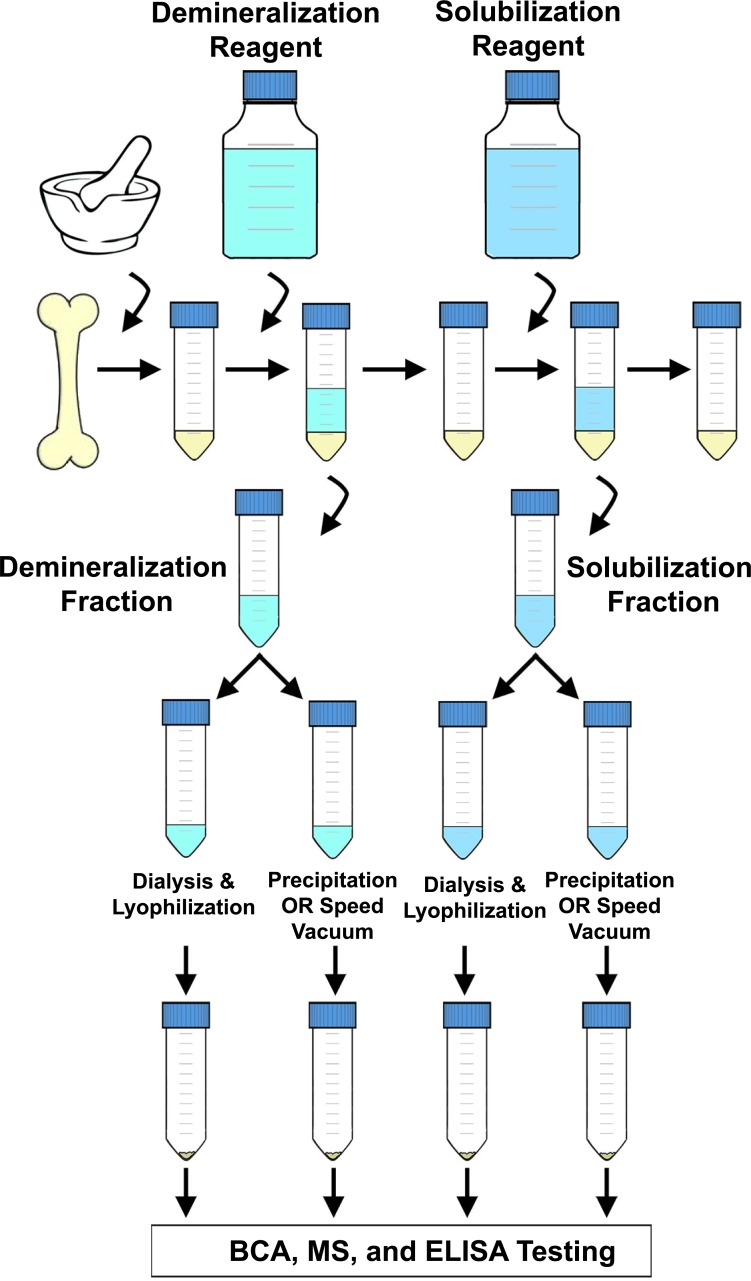
Flow chart depicting a generalized extraction method. Chicken bone was ground, placed in a centrifuge tube, and then incubated with a demineralizing reagent. After a period of incubation, the demineralizing reagent was removed and collected, and a solubilization reagent added. After another incubation period, the solubilizing reagent was removed and collected, and the pellet was discarded. Each supernatant was then split between two different buffer removal methods (e.g., precipitation, dialysis and lyophilization, drying by speed vacuum) and the subsequent extraction products were then kept separate for BCA, MS, and ELISA analyses.

To compare the efficiency of separate parts of extraction methods in depth, we applied seven protocols that use a variety of reagents to demineralize bone mineral and solubilize remaining matrix proteins (Table 1) to fresh chicken bone. Although the literature describes a plethora of bone protein extraction procedures, the methods we selected reflect similar protocols that have previously been used or suggested for *fossil* bone (e.g., Buckley et al., 2009; Buckley et al., 2010; Buckley & Wadsworth, 2014; Cleland, Voegele & Schweitzer, 2012; Schweitzer et al., 2007; Schweitzer et al., 2009; Wadsworth & Buckley, 2014). At each step, the collected supernatants (containing extracted proteins) were divided and subjected to two different methods to remove reagent buffers and concentrate extracted products. The experimental design, graphically represented in Fig. 1, resulted in 22 unique fractions representing varied demineralizing reagents, pretreatment of pellet prior to demineralization, volume and/or number of serial incubations of the same demineralizing reagent, solubilization reagents used in tandem with different demineralizing reagents, and buffer removal methods (e.g., precipitation, dialysis and lyophilization, or drying by speed vacuum). Each fraction was evaluated separately by bicinchoninic acid (BCA) protein assay, tandem MS/MS, and ELISA, allowing us to quantify differences in the amount of bulk bone protein extracted, the diversity of unique proteins identified, and the percent of peptide sequence recovered for select proteins (relative to the primary FASTA sequences listed in UniProt).

**Table 1:** Extraction protocols. Demineralization and solubilization reagents, as well as applied volumes, incubation times and temperatures, and any wash stages, are provided in standardized format for easy comparison. At the bottom of each treatment description is the identifying code for the precipitated and dialyzed portions of each fraction produced that is used throughout the manuscript. This code combines the reagent volume, any pretreatment before reagent incubation, reagent, and buffer removal method into a shortened form (e.g., 20-H/ABC-D).

Method	Demineralizing agent (mL solution/g of bone)	Solubilizing agent (mL solution/g of bone)	Special steps
**Method 1** HCl-GuHCl (Cleland, Voegele & Schweitzer, 2012; Wadsworth & Buckley, 2014)	0.6 M HCl 5 mL/g for 1 day @ RT **5-HCl-D** & **5-HCl-P**	4 M GuHCl in 0.05 M Tris 5 mL/g for 1 day @ 65 °C **5-H/GuHCl-D** & **5-H/GuHCl-P**	
**Method 2** HCl-SDS (Craig & Collins, 2002)	0.6 M HCl 5 mL/g for 1 day @ RT **5-HCl-D** & **5-HCl-P**	2% SDS 10 mL/g for 2 days @ RT 10 mL/g for 2 days @ RT **20-H/SDS-D** & **20-H/SDS-P**	Wash pellet with H_2_O after HCl
**Method 3** HCl-Urea (Rabilloud, 1998)	0.6 M HCl 5 mL/g for 1 day @ RT **5-HCl-D** & **5-HCl-P**	8 M Urea, 2 M Thiourea, 1% CHAPS, 0.05 DTT 10 mL/g for 2 days @ RT 10 mL/g for 2 days @ RT **20-H/Urea-D** & **20-H/Urea-P**	Wash pellet with H_2_O after HCl
**Method 4** HCl-ABC (Buckley et al., 2009; Buckley et al., 2010)	0.6 M HCl 20 mL/g for 1 day @ RT **20-HCl-D** & **20-HCl-P**	0.05 M (NH_4_)HCO_3_ 20 mL/g for 1 day @ 65 °C **20-H/ABC-D** & **20-H/ABC-SV**	Wash pellet with H_2_O after HCl
**Method 5** EDTA-GuHCl (Schweitzer et al., 2009)	0.5 M EDTA 4 mL/g for 1 day @ RT 2 mL/g for 5 days @ RT **6-EDTA-D**	6 M GuHCl in 0.1 M Tris 2 mL/g for 1 day @ 65 °C 2 mL/g for 1 day @ 65 °C 4 mL/g for 2 days @ 65 °C **8-E/GuHCl-D** & **8-E/GuHCl-P**	
**Method 6** EDTA-ABC- GuHCl	0.5 M EDTA 20 mL/g for 5 days @ 4 °C **20-EDTA-D**	0.05 M (NH_4_)HCO_3_ 20 mL/g for 1 day @ 65 °C **20-E/ABC-SV** 4 M GuHCl in 0.05 M Tris 20 mL/g for 1 day @ 65 °C **20-E/A/GuHCl-D**	
**Method 7** EDTA- Acetic Acid (Liu et al., 2012; Singh et al., 2011)	0.1 NaOH 20 mL/g for 4 h @ RT 0.5 M EDTA (1) 20 mL/g for 3 days @ 4 °C **20-N/EDTA1-D** 0.5 M EDTA (2) 20 mL/g for 3 days @ 4 °C **20-N/EDTA2-D**	10% Butyl Alcohol 20 mL/g for 1 day @ 4 °C 0.5 M Acetic Acid 15 mL/g for 3 days @ 4 °C **15-E/Acetic-D** 0.5 M Acetic Acid/w Pepsin 20 mL/g for 2 days @ 4 °C **20-E/A/Pepsin-D**	NaOH and butyl alcohol were discarded. Multiple H_2_O washes of pellet.

## Materials and Methods

### Sample preparation

Two samples were evaluated using each extraction method; one containing ground, defatted chicken bone, and one “blank” sample, consisting of extraction buffers (but no bone samples) to serve as a negative control. Chicken tibiotarsi were defleshed, then degreased whole in 10% Shout (Johnson Co.) with stirring for 1–2 days to remove lipids and blood from the medullary cavity and periosteal surfaces. Bones were then sectioned into large (∼1 cm) pieces and degreased for an additional day. Concentrated Shout contains small amounts (0.001% –1%) of the enzyme subtilisin, which has been shown to have collagen-cleaving properties (Ran et al., 2013); however we applied a diluted form the detergent (0.0001–0.1% enzyme), and only to whole bone pieces prior to grinding or homogenization. Thus, we think it is unlikely that the trace amounts of enzyme significantly affected the proteome of the tissue deeper in the cortex. After triple washing in 18.2 MΩ water to remove any remaining detergent from the bone surfaces, pieces were frozen with liquid nitrogen for 20 s and then ground with a mortar and pestle to the consistency of coarse sand. Bone powders from all bones were homogenized before aliquotting to ensure uniform samples of tissue for all extractions.

### Protein extractions

The basic steps and parameters of each protein extraction protocol are summarized in Table 1, and details of each method are provided below. For all protocols, “samples were centrifuged” refers to centrifugation at 7,200 rcf for 20 min at 4 °C unless otherwise stated. Volumes of reagents applied are given as “mL/g,” referring to the mL of reagent applied per gram of bone.

#### Method 1 (HCl–GuHCl)

Protocol modified from Cleland, Voegele & Schweitzer (2012) and Wadsworth & Buckley (2014). Bone powder (2 g) was demineralized in 0.6 M hydrochloric acid (HCl) (5 mL/g) and incubated at room temperature (RT) overnight with rocking. Samples were pelleted by centrifugation, and supernatants were collected. Pellets were resuspended in 5 mL/g 4 M guanidine hydrochloride in 50 mM Tris (pH 7.4), followed by incubation at 65 °C on a heating block overnight. After incubation, samples were centrifuged again, supernatants collected, and pellets discarded.

#### Method 2 (HCl–SDS)

Protocol modified from Craig & Collins (2002). Bone powder (2 g) was demineralized by incubation in 0.6 M HCl (5 mL/g) at RT overnight with rocking, centrifuged, and supernatants collected. Remaining pellets were neutralized by washing three times with 5 mL of 18.2 MΩ water, then resuspended in 10 mL/g of 2% sodium dodecyl sulfate (SDS) and incubated at RT with rocking for 2 days. Samples were centrifuged and supernatants were collected. The solubilization step was then repeated. SDS supernatants were combined and pellets were discarded.

#### Method 3 (HCl–Urea)

Protocol modified from Rabilloud (1998). Bone powder (2 g) was demineralized in 0.6 M HCl (5 mL/g) and incubated at RT overnight with rocking. Samples were centrifuged and supernatants were collected. Pellets were neutralized by washing 3 times with 5 mL of 18.2 MΩ water, and wash water was discarded. Pellets were resuspended in 10 mL/g of 8 M urea, 2 M thiourea, 1% CHAPS, and 0.05 M DTT (hereafter, “Urea”). After incubating at RT with gentle agitation for two days, samples were centrifuged, supernatants collected, and the solubilization step was repeated. Resulting supernatants were combined and pellets discarded.

#### Method 4 (HCl–ABC)

Protocol modified from Buckley et al. (2009) and Buckley et al. (2010). Bone powder (2 g) was demineralized in 0.6 M HCl (20 mL/g) and incubated at RT overnight as above. Samples were centrifuged and supernatants collected. Pellets were neutralized by washing six times with 20–40 mL 18.2 MΩ water until pH reached 6, and wash water was discarded. Pellets were resuspended in 20 mL/g of 50 mM ammonium bicarbonate (ABC) and incubated at 65 °C overnight in a heating block. Samples were centrifuged, supernatants collected, and pellets discarded.

#### Method 5 (EDTA–GuHCl)

Protocol modified from Schweitzer et al. (2009). Bone powder (2 g) was demineralized in 0.5 M ethylenediaminetetraacetic acid (pH 8.0) (EDTA) (4 mL/g) and incubated at RT overnight with rocking. Samples were centrifuged at 6,000 rcf for 15 min and supernatants were collected. Pellets were resuspended in an additional 2 mL/g 0.5 M EDTA, incubated at RT for 5 days with rocking, and then centrifuged. EDTA supernatants were collected and pooled with the previous EDTA fraction. Pellets were resuspended in 2 mL/g of 6 M guanidine hydrochloride in 0.1 M Tris (pH 7.4) and incubated at 65 °C on a heating block overnight. Samples were centrifuged and supernatants were collected. The solubilization step was repeated twice more (again with 2 mL/g for 1 day, then with 4 mL/g for 2 days), and all three GuHCl supernatants were combined (total final volume 8 mL/g). Pellets were then discarded.

#### Method 6 (EDTA–ABC–GuHCl)

For this extraction, only 1 g of bone sample was prepared, because subsequent fractions were not split between post-processing methods (see below). Bone powder (1 g) was demineralized in 0.5 M EDTA (pH 8.0) (20 mL/g) incubated at 4 °C for 5 days with rocking. Samples were centrifuged and supernatants were collected. Pellets were resuspended in 20 mL/g of 50 mM ABC, incubated at 65 °C overnight, then centrifuged and supernatants collected. Pellets were resuspended in 20 mL/g of 4 M GuHCl in 50 mM Tris (pH 7.4) and incubated at 65 °C overnight. Samples were centrifuged, the final supernatants were collected, and pellets were discarded.

#### Method 7 (EDTA–Acetic acid)

Collagen purification protocol modified from Liu et al. (2012) and Singh et al. (2011). This narrowly targeted protocol is used to purify collagen I for inoculation; it was included to compare its efficacy for collagen I coverage against more generalized extraction methods. To remove NCPs, bone powder (2 g) was incubated in 0.1 M sodium hydroxide (NaOH) (20 mL/g) at 4 °C for 4 h with rocking. Samples were centrifuged and supernatants were discarded. Pellets were washed six times with 20 mL of 18.2 MΩ water, until wash water reached a neutral pH, and wash water was discarded. To demineralize, 20 mL/g of 0.5 M EDTA (pH 8.0) was added to each pellet and incubated at 4 °C with rocking for three days. Samples were centrifuged and supernatants collected. The demineralization step was repeated, and subsequent supernatants were kept separate for analysis. Pellets were washed with 20 mL 18.2 MΩ water 5 times, then incubated in 20 mL/g 10% butyl alcohol overnight at 4 °C to remove any fats remaining after Shout pretreatment of the periosteal and medullary bone surfaces (see above). Samples were centrifuged and supernatants discarded. Pellets were washed as above, then incubated in 15 mL/g 0.5 M acetic acid at 4 °C for three days to solubilize liberated collagen. Samples were centrifuged and supernatants collected. Pellets were resuspended in 20 mL/g 0.5 M acetic acid with 500 µg/mL pepsin A for two days at 4 °C. Samples were centrifuged, supernatants were collected, and remaining pellets were discarded.

Acetic acid fractions were filtered through densely packed fiberglass (Pyrex) in a 10 mL syringe. The acetic acid fraction without pepsin was neutralized with NaOH, then supernatants were brought to a 0.9 M NaCl concentration and incubated overnight at RT. Samples were centrifuged at 7,200 rcf for 40 min. Resulting supernatants were transferred to new tubes, and pellets were stored at 4 °C. NaCl was added to the supernatants to a final concentration of 2.6 M and samples were incubated overnight at RT. Samples were again centrifuged at 7,200 rcf for 40 min and supernatants were discarded. Pellets (0.9 M and 2.6 M) were combined and resuspended in 20 mL 0.5 M acetic acid, then salted to 2.0 M and again incubated overnight at RT. Samples were centrifuged as above, supernatants discarded, and pellets resuspended in 20 mL 0.5 M acetic acid. Acetic acid fractions with pepsin were prepared without neutralizing the pellet. Supernatants were brought to 2.0 M NaCl and incubated overnight at RT. Samples were centrifuged as above, supernatants discarded, and the pellets resuspended in 20 mL 0.5 M acetic acid. The 2.0 M salt-out step was repeated, and the final pellets were resuspended in 20 mL 0.5 M acetic acid.

### Buffer and salt removal and concentration of proteins

After extractions were performed and supernatants collected, different post-extraction desalting and buffer removal methods were employed to test the efficacy of each for removing buffers, bone minerals, residual salts, detergents, and other non-protein extraction products that can interfere with downstream analyses (e.g., pellet weight calculation, protein quantification assays, ionization in MS) (Visser, Lingeman & Irth, 2005) while retaining protein. All extraction supernatants collected were centrifuged to pellet any debris, then divided equally into two fractions, each placed into pre-weighed tubes and stored at 4 °C. For all fractions of all protocols, half of the extraction supernatant was dialyzed and lyophilized to dryness, and half was precipitated, except as noted below. At the end of each method, post-weighing sample tubes allowed for calculation of pellet mass.

#### Precipitation

To HCl fractions, 1 mL of 100% trichloroacetic acid (Rajalingam et al., 2009) was added per 4 mL of HCl, followed by overnight incubation at 4 °C, centrifugation, and discarding of supernatants. Remaining pellets were washed twice with 5 mL of 100% cold acetone. To GuHCl and SDS fractions, 5 mL of 100% ethanol was added per 1 mL of supernatant, followed by overnight incubation at −20 °C. Samples were centrifuged, supernatants were discarded, and remaining pellets were washed twice with 90% ethanol. To Urea fractions, 5 mL of 100% cold acetone was added per 1 mL of Urea and samples were incubated overnight at −20 °C. Samples were centrifuged and supernatants were discarded. Remaining pellets were washed twice with 5 mL 100% cold acetone. All tubes were inverted and pellets were allowed to dry at RT.

Note: EDTA, ABC, and acetic acid fractions were not subject to precipitation; all EDTA fractions were dialyzed, ABC fractions were split between drying by speed vacuum and dialysis/lyophilization (see below), and acetic acid fractions specific treatment (see below).

#### Dialysis

EDTA, HCl, SDS, ABC, Urea, GuHCl, and Acetic Acid supernatants were placed into 3,500 MWCO SnakeSkin^®^ dialysis tubing (Thermo Scientific) and dialyzed against 4 L of 18.2 MΩ water (acetic acid fractions were dialyzed against 0.1 M acetic acid) for 4 days at 4 °C, exchanging dialysis water two times daily. After the final exchange, dialyzed samples were aliquoted into pre-weighed centrifugation tubes, frozen at −80 °C, and lyophilized to completion for 2–5 days.

#### Drying by speed vacuum

Half of the ABC fractions collected were transferred directly into pre-weighed tubes and dried to completion using a speed vacuum (instead of precipitation). Because speed vacuuming is a technique that has been used for removal of ABC in previous bone protein extraction experiments (e.g., Cleland, Voegele & Schweitzer, 2012), analysis of this treatment is vital. Therefore, to accommodate the experimental design (which requires each extraction supernatant to be split in half), we substituted speed vacuuming as one of the two buffer removal methods for ABC samples alongside dialysis and lyophilization, and excluded precipitation.

Subsequent to all buffer removal methods, final pelleted extraction products were weighed. Weighed aliquots of each pellet were then solubilized in 1.5 mL 50 mM ABC so that precise quantities of extraction products could be easily and consistently measured for future assays. Prior to each assay, sample aliquots were dried by speed vacuum before solubilization in assay buffer.

For clarity, individual fractions are hereafter referred to by a unique identifier code that includes the volume of reagent applied per gram of bone (e.g., 5 mL/g, 20 mL/g), the reagent applied (e.g., HCl, EDTA), and the buffer removal method used (e.g., precipitation, dialysis). For example, 5 mL/g HCl fractions that were precipitated are “5-HCl-P,” and dialyzed 20 mL/g ABC fractions, collected after demineralization with EDTA, are “20-E/ABC-D.” These codes are listed in Table 1.

### Bicinchoninic acid (BCA) protein assay

To determine total protein content in the final, post-lyophilization or precipitation pellet, a Pierce BCA Protein Assay Kit (Thermo Scientific) was used according to the manufacturer’s specifications (Thermo-Scientific, 2002). Aliquots (25 µg) of lyophilized, precipitated, or dried (by speed vacuum) extract from each fraction (see above) were resolubilized in 25 µL phosphate buffered saline solution (PBS) and plated (in duplicate) on a 96 well microtiter plate. 200 µL of working reagent (50 parts BCA kit reagent A to 1 part reagent B) were added to all wells, and plates were incubated at 37 °C for 30 min. Absorbance was measured on a Multiskan Spectrum 1500 microplate reader (Thermo Scientific) at a wavelength of 562 nm using SkanIt 2.2 software. Sample protein content was determined against a standard absorbance curve calculated from a serial dilution of bovine serum albumin (BSA) in PBS to the following concentrations (25 µL plated per well): 2 mg/mL, 1.5 mg/mL, 1 mg/mL, 750 µg/mL, 500 µg/mL, 250 µg/mL, 125 µg/mL, 25 µg/mL, and 0 µg/mL (PBS only). Assays were repeated 4–7 times for each sample and total protein content per 25 µg of pellet was assessed as the average of these multiple trials. Overall protein recovery and pellet purity were calculated based on these results and the total weight of the recovered pellet for each fraction. Extract products from “buffer only” samples were also tested to confirm that interference from left-over reagents did not cause spuriously high results in bone samples.

### Protein digestion and mass spectrometry

Duplicate aliquots of 20 µg protein were prepared from the following fractions: all fractions of Methods 1 and 4–6, the solubilization fractions of Methods 2 and 3 (dialysis only of 3), and the demineralization fractions of Method 7. Methods that had fractions identical to some already tested (HCl from 2 and 3), extreme amounts of non-protein precipitation in the blank controls (precipitated “Urea” from 3), or solubilization fractions with extremely low amounts of total protein yield (acetic acids from 7) were excluded. Samples were reduced in 20 µL of 8 M urea (previously deionized with BioRad AG 501-X8 resin according to the manufacturer’s protocol) and 2 µL of 100 mM DTT diluted in 100 mM ABC (final concentration ∼9.1 mM DTT) for 20 min at RT, then alkylated with 3 µL of 300 mM iodoacetamide diluted in 100 mM ABC (final concentration ∼32.14 mM iodoacetamide) for 30 min at RT in the dark. Proteins were digested overnight with 200 ng of Trypsin Gold (mass spectrometry grade; Promega, activated for 15 min at 30 °C prior to application) diluted with 135 µL of 100 mM ABC and 1 µL of 1 M DTT. Digestion was terminated with 2 µL of 100% formic acid (FA) and resulting peptides were desalted and concentrated with C18 ZipTips (Millipore) as follows: Tips were activated with 90% acetonitrile (ACN), 0.2% FA, and equilibrated with 0.2% FA. Peptides were bound to tips, then washed with 0.2% FA and eluted into 30 µL of 70% ACN, 0.2% FA. Samples were dried by speed vacuum to remove acetonitrile, then stored at −80 °C until analysis.

Samples were resuspended in 27 µL of “Buffer A” (95% Optima grade water (Fisher Scientific), 5% Optima grade ACN (Fisher Scientific), 0.2% FA), centrifuged at 21,000 × g for 10 min at 4 °C, and transferred to autosampler vials (RSA™ AQ™; MicroSolv) with caps containing PFTE septa (Fisher Scientific). A Dionex Ultimate 3000 UHPLC System (Thermo Scientific) injected 6 µL of each sample onto a self-packed C18 Aqua (3 µm particle size, 125 Å pore size; Phenomenex) trap column (4 cm L, 150 µm ID). Peptides were washed and desalted for 10 min at a rate of 2.5 µL/min, then transferred to and eluted from a self-packed C18 Aqua analytical column (20 cm L, 75 µm ID) and spray emitter (12 cm L, 15 µm ID, New Objective, self-packed with 2 mm C18 Aqua resin) at 300 nL/min with the following gradient: 5% B at 0 min, 5% B at 12 min, 40% B at 45 min, 85% B at 47 min, 85% B at 49 min, 5% B at 51 min., and 5% B at 60 min. (A: 95% Optima grade water (Fisher Scientific), 5% Optima grade ACN (Fisher Scientific), 0.2% FA; B: 95% Optima grade ACN, 5% Optima grade water, 0.2% FA). Eluted peptides were then introduced into a custom 12T LTQ Velos FT-ICR mass spectrometer (Thermo Scientific) for analysis by tandem MS/MS. Full-scan FT MS1 spectra were obtained with a 400–1800 *m*∕*z* scan range at a resolving power of 85.7k. The top 8 most abundant peaks per MS1 scan were then selected for fragmentation by collision-induced dissociation (CID). MS2 scans were performed in the ICR cell (FT/FT) in centroid mode with an isolation window of 4 *m*∕*z*, a normalized collision energy of 35%, an activation *q* of 0.25, and a duration of 15.0 ms at a resolving power of 42.9k. Dynamic exclusion was enabled with the following parameters: repeat count of 2, repeat duration of 45 s, exclusion duration of 30 s, and an exclusion list size of 500. Total instrument acquisition time was 50 min for each sample run.

### Data analysis

Spectra were searched in PEAKS (version 7.5; Bioinformatics Solutions Inc.) using a precursor mass tolerance of 10 ppm and a fragment ion mass tolerance of 0.2 Da. Two missed cleavages were allowed, as well as nonspecific cleavage at one end of the peptide. The following post translational modifications were allowed: fixed—carbamidomethylation [C]; variable—oxidation [M], oxidation or hydroxylation [RYFPNKD], [G]@C terminal, pyro-glu from Q, and deamidation [NQ]. Spectra were searched against the UniProt_chicken database, and PEAKS PTM and SPIDER were enabled to account for unspecified post-translational modifications (PTMs) or mutations, respectively. Results were filtered using ≤1% FDR for peptide spectral matches (PSMs), and either a protein score of −log_10_*p* ≥ 20, or ≤1% FDR (whichever was more stringent) plus at least 1 unique peptide for proteins. Effective FDR values and associated expect score cut-offs for each file searched, as well as the number of MS and MS/MS scans performed, is provided in Table S1. Search results were analyzed by hand: peptides corresponding to common lab contaminants (e.g., keratin) were eliminated from consideration, and duplicate matches of one spectrum to multiple protein accession IDs were reduced. Additionally, proteins identified by only 1 peptide in 1 injection were eliminated (23 proteins), and only those found in multiple injections, or as multiple peptides from a single injection, were used for comparison. Finally, data from duplicate trials of the same fractions were combined to account for variation between trials.

### Enzyme-linked immunosorbent assay (ELISA)

After total pellet protein concentration was calculated (see methods), aliquots of protein extract from each pellet were solubilized with PBS into 10 µg/mL solutions. 100 µL aliquots of sample solutions (1 µg of extracted bone protein) or 100 µL of PBS were added to the wells of an immulon U-bottom 96-well microtiter plate (Thermo Scientific) and allowed to incubate for 1–4 h at RT. After removal of antigen, to prevent non-specific binding of antibodies directly to the plate, wells were blocked with 200 µL of 5% BSA diluted in PBS with Tween20 and thimerosal (hereafter “5% BSA”) and the plate was incubated either 1–3 h at RT or overnight at 4 °C. A portion of wells from each sample was then incubated with 100 µL of the following treatments, diluted in 5% BSA: polyclonal chicken specific anti-collagen I (US Biological, C7510-13B) diluted 1:1000, polyclonal anti-alligator hemoglobin (Bio-Synthesis, Inc., host # BYSN 6941, lot # AB1421-3) diluted to 1:700 or 1:850, or 5% BSA only. We applied antibodies raised against alligator hemoglobin (HB), rather than commercially available polyclonal antibodies raised against chicken HB, because: (1) they were readily available in the time allotted for the experiment; (2) previous tests on antibodies against chicken HB were found to demonstrate non-specific binding to multiple proteins (M Schweitzer, 2006, unpublished data); (3) chicken and alligators are both archosaurian taxa (Brochu, 2001), therefore, substantial overlap in their hemoglobin epitopes is predicted.

Plates were incubated 1–3 h at RT or overnight at 4 °C, then washed 20 times in ELISA wash buffer (10% PBS diluted 18.2 MΩ water with Tween20). Wells received 100 µL of secondary antibody (alkaline phosphatase conjugated goat anti-rabbit IgG (H + L)) diluted 1:2000 in PBS and were incubated 1–2 h at RT, then washed an additional 20 times with ELISA wash buffer. 100 µL of substrate (diethanolamine substrate buffer + p-Nitrophenylphosphate, Pierce PNPP Kit) was added to each well, and absorbance at 405 nm was measured with a microplate reader (Multiskan Spectrum 1500; Thermo Scientific). Data were acquired using SkanIt 2.2 software. Measurements were taken at time = 0, 10, 20, 30, 40, 60, 90, 120, 150, 180, 210, 240, and 270 min.

## Results

### BCA protein assay

The purity of the final pellet extracted from each method after precipitation, dialysis and lyophilization, or speed vacuuming, and thus the total recovered protein for each sub-extraction, was determined by BCA assay. Graphs of the final pellet weight compared to the calculated total protein recovered are shown in Figs. S1A and S1B. In every instance, dry pellet weight did not reflect actual extracted protein yield; total pellet weights were up to 15 times greater than the weights of their calculated protein content. The maximum purity of any pellet in this study (i.e., percent of total pellet weight that was protein) was 44.8% in 20-H/Urea-D, followed by 43.8% in 20-H/ABC-SV. Significantly, all extractions and post-extraction buffer removal methods conducted here produced a pellet that comprised more than 50% non-protein solids by weight.

The total protein yields per gram of bone analyzed for each demineralization and solubilization fraction were calculated from the BCA results and are shown in Figs. S2A and S2B, respectively. Demineralization fractions (Fig. S2A) recovered 0.51–6.3 mg protein per gram of bone analyzed. The highest recovery was observed from 20-N/EDTA-D (6.30 mg/g), followed by 20-HCl (whether precipitated or dialyzed) (4.59–4.74 mg/g), 20-EDTA-D (4.30 mg/g), 8-EDTA-D (2.77 mg/g), 5-HCl (whether precipitated or dialyzed, 0.74–1.03 mg/g), and finally, a second serial incubation of 20-N/EDTA2-D (0.51 mg/g).

Solubilization fractions (Fig. S2B) recovered between 0–58.39 mg of protein per gram of bone analyzed; the highest recovery values in the solubilization fractions exceeded that of the demineralization fractions by an order of magnitude. 20-ABC-SV recovered the most protein regardless of demineralizing agent (51.59–58.39 mg/g), followed by 20-H/ABC-D and GuHCl regardless of demineralizing agent, buffer removal method, or molar concentration (22.77–28.17 mg/g). Additionally, 20-E/A/GuHCl-D recovered 13.76 mg/g protein *after* an ABC solubilization step (Method 6). This was similar to 20-H/Urea extractions, which recovered 11.78–13.5 mg/g of protein regardless of buffer removal method, although an anomalously high amount of non-protein precipitant interference in the blank, buffer-only samples was observed (see below). 20-H/SDS-D recovered 8.08 mg/g, and 20-H/SDS-P less than a quarter that amount (1.52 mg/g). Acetic acid yields were below the limits of detection for this assay.

### Mass spectrometry

For full consideration of their utility to bone proteomic research, mass spectrometry data obtained from all fractions were analyzed in three different contexts: (1) quantity of peptide-spectral matches (PSMs)/peptides recovered; (2) diversity of identified proteins and the redundancy of protein identifications between fractions within a method; and (3) the depth of sequence coverage (i.e., percent of recovered sequence length vs. total sequence length for select proteins).

#### PSMs/peptides

**Figure 2:**
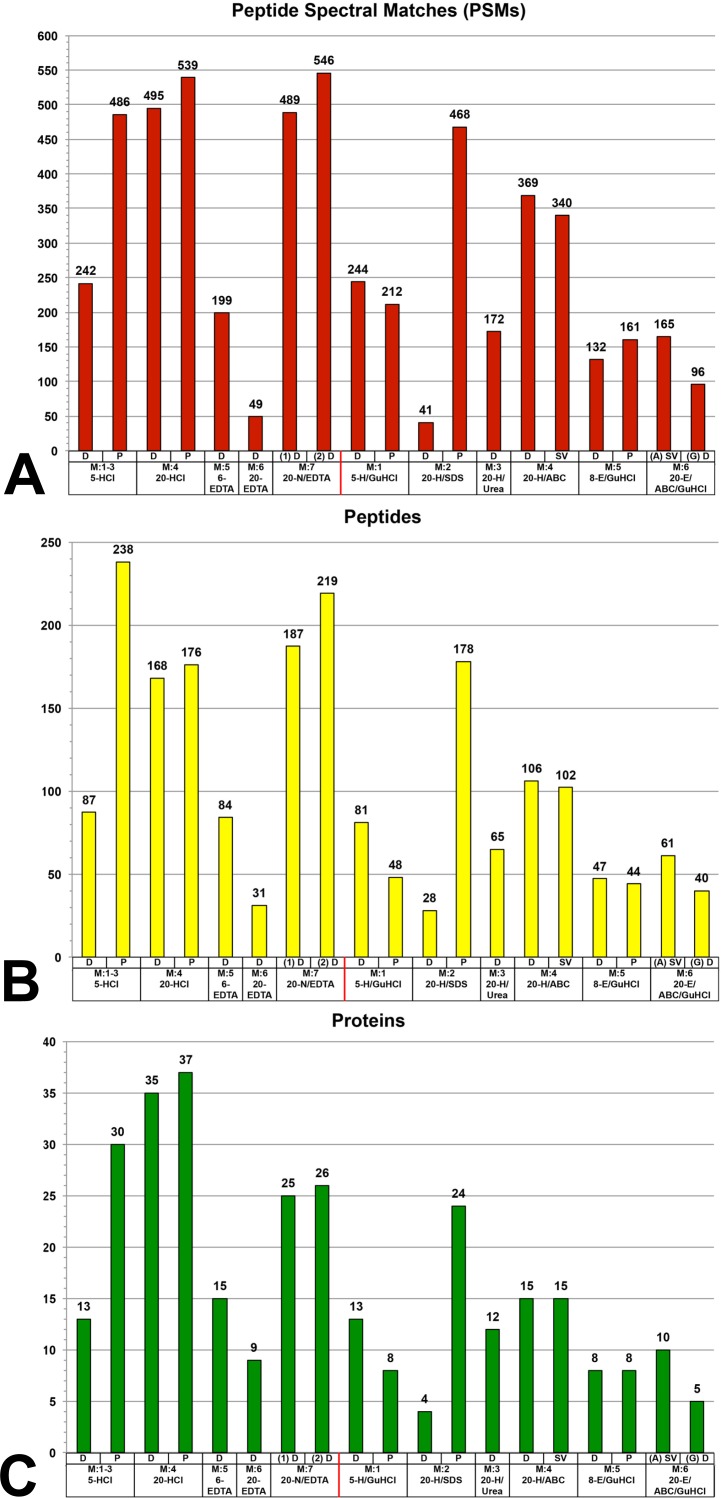
Graphs of the total number of (A) peptide spectral matches (PSMs), (B) unique peptides (PTM variations eliminated), and (C) proteins identified within each fraction evaluated by mass spectrometry. (A) The greatest number of PSMs were recovered mainly from demineralization fractions, specifically 20-HCl fractions, 5-HCl-P, and 20-N/EDTA-D fractions, which all recovered 550–450 PSMs. The only solubilization fraction to recover PSMs within this range was 20-H/SDS-P. The next highest values were achieved by 20-H/ABC, whether dialyzed or dried by speed vacuum. This pattern was generally the same for total number of peptides (B), though 5-HCl-P had a greater diversity of unique peptides than higher volume (20-HCl) fractions, which had a greater number of PSMs. This relative pattern was again repeated in the numbers of unique proteins identified (C), which show that HCl, NaOH treated EDTA, and precipitated SDS recovered broader portions of the bone proteome than other extraction steps.

The highest number of PSMs (450–550) were recovered primarily from demineralization fractions (Fig. 2A), specifically 20-HCl-D, 20-HCl-P, 5-HCl-P and both 20-N/EDTA-D fractions. The one solubilization fraction that was in this range was 20-H/SDS-P, which recovered 468 PSMs. 20-H/ABC recovered ∼350 PSMs whether dialyzed or dried by speed vacuum. All other fractions recovered less than 250 PSMs; the poorest overall recovery was observed in 20-EDTA-D and 20-H/SDS-D fractions (<50 PSMs).

When only unique peptides are analyzed (i.e., when PSM duplicates and PTM variation are reduced) (Fig. 2B), demineralization fractions recovered the highest numbers of peptides (∼150–250), including 20-HCl fractions, 5-HCl-P, and both 20-N/EDTA-D fractions, with 5-HCl-P recovering the greatest number overall (238 peptides). The only solubilization fraction that recovered a total number of peptides within this range was 20-H/SDS-P, with 178 peptides. 5-HCl-D, 6-EDTA-D, 5-H/GuHCl-D, 20-H/Urea-D and 20-ABC (all variants) recovered 50–150 peptides. All remaining fractions recovered less than 50 unique peptides across two combined proteomic analyses.

#### Protein diversity and redundancy

A total of 55 unique proteins (identified from at least two unique peptides) were recovered across all fractions (Table 2). Of the 55 proteins identified in this study, 14 had accession numbers that corresponded to “uncharacterized” proteins. However, these proteins all contained substantial overlap with bone proteins from other avians when searched against SwissProt using UniProt’s basic local alignment search tool (BLAST) (Liu et al., 2012). For ease of discussion, these “uncharacterized” proteins will be referred to by their matches in BLAST, marked with brackets to differentiate them from the fully characterized proteins that were also recovered (e.g., [kininogen], [Pigment epithelium derived factor (PEDF)]). A full list of proteins identified with their corresponding accession numbers, protein descriptions, and BLAST matches is provided in Table S2.

**Table 2:** Breakdown of proteins identified in each fraction, and the number of peptides recovered for each (including variations in PTMs). Demineralization and solubilization fractions are divided by the centerline. Proteins for which 5+ peptides were recovered in any fraction are bolded. Of these proteins, fractions that resulted in the most peptide identifications are marked in dark purple, and fractions with peptide identifications within one standard deviation of the highest value are marked in light purple. The highest diversity in identified proteins was observed predominantly in the demineralization fractions (left), which also resulted in the greatest numbers of peptides from most proteins. One notable exception was collagen I, alpha 2; various solubilization fractions (e.g., ABC, GuHCl) resulted in more peptide identifications for collagen I, alpha 2 than found in any demineralization fraction.

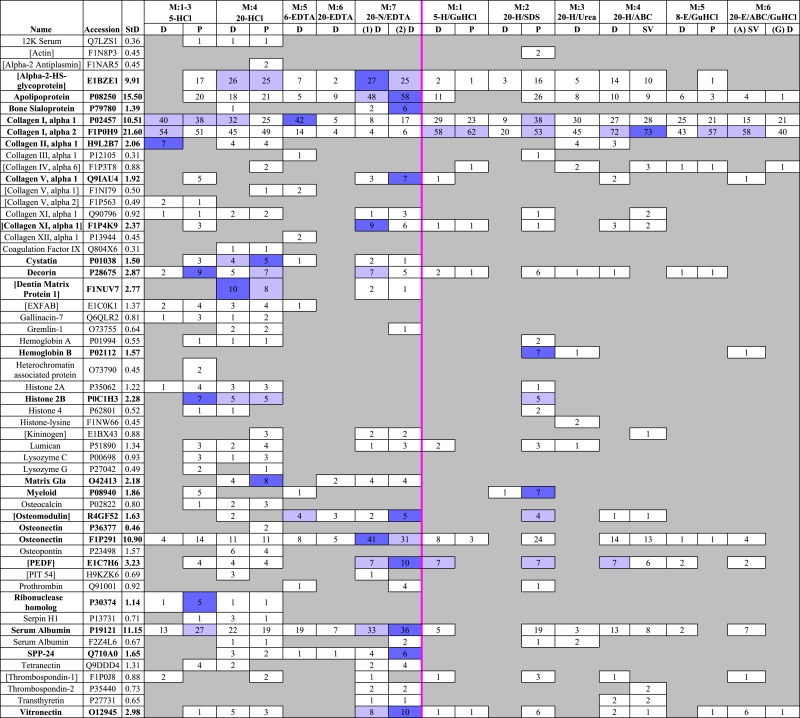

The greatest number of proteins was identified in 20-HCl-P, which recovered 37 proteins (Fig. 2C). Demineralization fractions accounted for the top five recoveries ranging from 25–37 proteins, including both 20-HCl-P and 20-HCl-D, 5-HCl-P, and both 20-N/EDTA1-D and 20-N/EDTA2-D. 20-H/SDS-P recovered 24 proteins, and was the only solubilization fraction in which more than 15 proteins were identified. All other fractions recovered fewer than 15 proteins.

**Figure 3:**
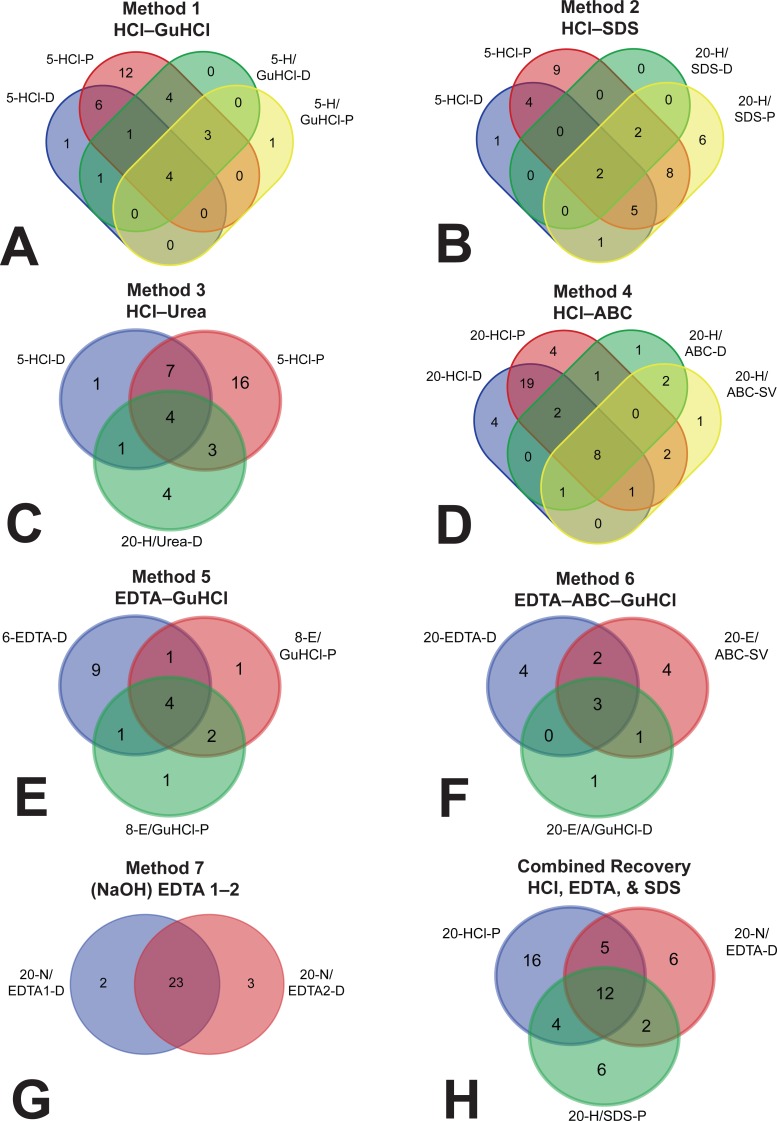
Venn diagrams depicting overlap (i.e., redundancy) of identified proteins between separate fractions. (A) Method 1, (B) Method 2, (C) Method 3, (D) Method 4, (E) Method 5, (F) Method 6, (G) demineralization fractions of Method 7, and (H) a hypothetical method that combines three fractions to maximize recovery. In methods 1–5 (A–E), there was a large amount of overlap between the demineralization and solubilization fractions, and most of the unique proteins were found in the demineralization fractions,rendering the solubilization fractions largely redundant. In Method 6 (F), there were slightly more unique proteins in the two solubilization fractions (combined) than in the one demineralization fraction. In Method 7, there was substantial redundancy in the two sequential demineralization incubations, indicating that additional demineralization did not recover substantially new portions of the proteome. Combining three fractions (i.e., 20-HCl-P, 20-N/EDTA-D, and 20-H/SDS-P) into a hypothetical extraction (H) accounted for 51 of the 55 proteins identified across all fractions in this study, with the largest contribution of unique proteins coming from HCl.

To investigate whether demineralization fractions were recovering a set of proteins distinct from those obtained in the subsequent solubilization fractions, we constructed Venn diagrams from the lists of proteins identified by each method (Fig. 3). In five of the methods, more than 60.0% of the proteins found in the solubilization fractions were redundant with proteins previously identified in the demineralization fractions (60.0–92.9%, Table S3). Method 6, which included only one demineralization fraction (20-EDTA-D) but two serial solubilization fractions (20-E/ABC-SV and 20-E/A/GuHCl-D), showed 45.5% redundancy when the two solubilization fractions were combined (5/11 proteins). Because of their minute yield, solubilization fractions for Method 7 were not tested by MS, and therefore we cannot assess their redundancy. However, the two serial incubations of demineralizing agent in Method 7 (20-N/EDTA1-D and 20-N/EDTA2-D) were very redundant; of the proteins recovered by each (25 in the first, 26 in the second), 23 were the same (92.0% and 88.5% redundancy, respectively).

#### Sequence coverage

To compare sequence coverage of the most abundant proteins, we ranked the proteins with the highest coverage in any fraction, then generated a heat map (Table 3) of the top 28 (i.e., top half) that compares their coverage in all fractions. Of these 28 proteins, demineralized fractions possessed the highest (or equal to highest) coverage for 23 proteins. 20-HCl-P gave the best (or equal to best) coverage for 9 proteins (12K serum protein, 45.7%; cystatin, 39.6%; decorin, 31.1%; [dentin matrix protein 1], 18.9%; gremlin-1, 16.8%; histone 2A, 30.2%; lumican, 17.5%; matrix Gla, 54.5%; osteocalcin, 29.9%) and 20-HCl-D for five proteins (12K serum protein, 45.7%; [alpha-2-HS-glycoprotein], 59.6%; cystatin, 39.6%; [dentin matrix protein 1], 18.9%; osteocalcin, 29.9%). Lower volume, 5-HCl-P obtained the best or equal coverage of eight proteins (12K serum protein, 45.6%; [EXFAB], 39.3%; gallinacin-7, 61.2%; histone 2B, 20.6%; lysozyme C, 34.7%; myeloid protein, 37.1%; ribonuclease homolog, 18.0%; tetranectin, 38.8%). High volume 20-N/EDTA1-D gave the best/equal coverage for one protein in the first incubation (osteonectin (F1P291), 69.8%) and five additional proteins in the second, 20-N/EDTA2-D (apolipoprotein, 70.8%; [PEDF], 34.4%; serum albumin (P19121), 51.2%; secreted phosphoprotein 24 (SPP-24), 25.0%; vitronectin, 24.1%). Solubilization fractions obtained the best/equal coverage for only six proteins—20-H/SDS-P for (collagen I alpha 1, 23.8%; hemoglobin beta, 36.1%; histone 4, 17.5%; lumican, 17.5%) and 20-H/ABC-SV for (collagen I alpha 2, 43.7%; transthyretin, 37.3%). The most abundant proteins in bone, collagen I alpha 1 and 2, showed a generally similar range of coverage across the board (9.2–23.8% and 23.0–43.7%, respectively), with the exception of EDTA fractions, which obtained coverage ranges of 4.7%–20.3% for collagen I alpha 1 and 6.8–13.5% for alpha 2.

**Table 3:** Heat map of the degree of peptide coverage obtained in all fractions for top 28 (top half) proteins with the highest coverage in any fraction. Demineralization and solubilization fractions are divided by the centerline. Of the 28 proteins listed, the greatest (or equal to greatest) degree of peptide coverage for 23 of them was detected in demineralization fractions. Notable exceptions to this trend include collagen I alpha 1 and alpha 2; best coverage for these abundant bone proteins was obtained in 20-H/SDS-P and 20-H/ABC-SV, respectively. A color legend for the heat map is provided at the bottom of the table.

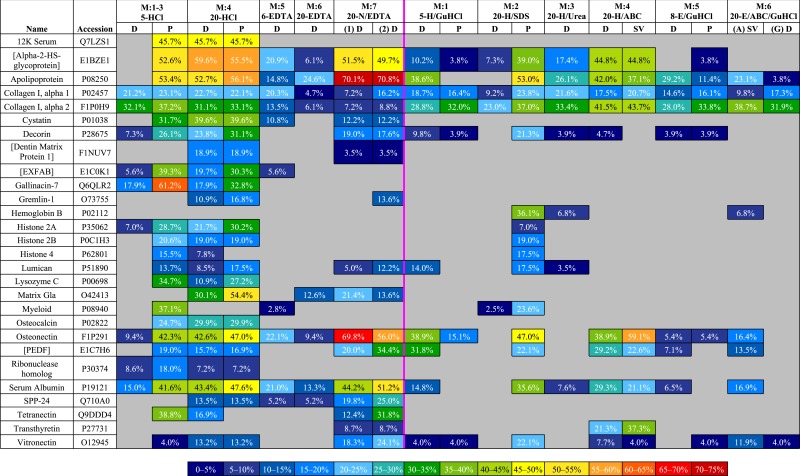

### ELISA

ELISA testing was performed to independently confirm select patterns observed in mass spectrometry results. ELISA assays plated with extracts from demineralization fractions as the antigen (Method 6 not included) with polyclonal anti-chicken collagen antibodies (Fig. S3A) yielded absorbance values as follows: 5-HCl fractions showed the highest absorbance values (0.78–0.84), which were similar (*p* = 0.1754) regardless of whether demineralization or precipitation was used as a buffer removal method. 20-HCl-D showed an absorbance value similar to that of 5-HCl fractions (0.74, *p* = 0.1342, 0.2346), but 20-HCl-P was significantly lower (0.58, *p* < 0.05). All EDTA fractions (20-N/EDTA1-D, 20-N/EDTA2-D, and 6-EDTA-D) showed the lowest absorbance values (0.46, 0.46, and 0.50 respectively), and were significantly lower than the values obtained for 5-HCl fractions and 20-HCl-D (*p* < 0.05 in all instances). A table of *p*-values for *t*-test results between all tested fractions is available in Table S4A.

To confirm the comparative efficiency of SDS for hemoglobin extraction, we tested a variety of extracts in ELISA with anti-alligator hemoglobin antibodies. ELISA performed on demineralization (Fig. S3B) and solubilization (Fig. S3C) fractions (Method 6 not included) with polyclonal antibodies raised against affinity purified alligator hemoglobin yielded absorbance values as follows: In the demineralization fractions (tested at an antibody concentration of 1:850), very low (but positive, as defined by absorbance at least twice above background levels (sensu Appiah et al., 2012; Ostlund et al., 2001; Tabatabai & Deyoe, 1984) absorbance values were obtained for HCl fractions 20-HCl-D, 20-HCl-P, and 5-HCl-P (0.02–0.09) and EDTA fractions 6-EDTA-D, 20-N/EDTA1-D, and 20-N/EDTA2-D (0.12–0.09). In the solubilization fractions (tested at an antibody concentration of 1:700), 20-H/Urea-D had very low, but positive absorbance (0.02). SDS fractions obtained the highest absorbance values, which were significantly greater (*p* < 0.05) than all other extracts tested; additionally, 20-H/SDS-D absorbance values (0.75) were three times that of 20-H/SDS-P (0.26). A table of *p*-values for *t*-test results between all tested fractions is available in Table S4B.

## Discussion

### Comparison of chicken extracts

The data generated by this study reveal a number of patterns that influence proteomic studies of bone. First, the dry weight of the pellet remaining after extraction and buffer removal did not accurately reflect its calculated protein content, in most cases by a very wide margin, and is therefore a poor indicator of actual protein yield (Fig. S1). In fact, pellet weight did not accurately predict *relative* protein content when comparing various extractions, in that larger pellets did not necessarily correspond to a larger protein yield. For example, 5-H/GuHCl-P had a pellet weight of 151.1 mg and a protein content of 28.15 mg, whereas 8-E/GuHCl-P produced a far larger pellet (357.3 mg) but less protein (23.48 mg) (Fig. S1B). In all cases, less than half the pellet mass could be ascribed to proteins, indicating that none of the buffer removal methods we employed completely removed salts, detergents, and other non-protein solids from the sample. The inaccuracy and inconsistency of the relationship between pellet weight and protein content suggest that pellet weight should not be used as a proxy for assessing the yield of a bone protein extraction or for preparing subsequent samples for further proteomic analyses.

The most important pattern shown by this data is that, although solubilization fractions (e.g., ABC, GuHCl) overall recovered far more bulk protein than demineralization fractions (e.g., HCl, EDTA) (Fig. S2), demineralization fractions contained a greater *diversity* of proteins in each µg of recovered protein extract (Table S2). This translates to the recovery of more NCPs, and in many cases better peptide coverage of those NCPs. Indeed, in all but one instance, the greatest proteomic diversity in each method resulted from whichever reagent was applied to the pellet *first* (the exception is Method 6, in which 20-E/ABC-SV extracted 10 proteins compared to the nine extracted in the preceding 20-EDTA-D).

This is critical knowledge when designing studies to elucidate the bone proteome. Although the large disparity in protein yield between the two fractions would seem to make the loss from discarding the demineralizing reagents negligible, our proteomic data shows that these demineralization fractions contain an extractome with a much greater wealth of proteomic diversity per µg. Additionally, as mass spectrometry does not require large amounts of protein (but rather analyzes protein concentrations on the order of µg to ng), the relatively low total mass of protein extracted by demineralizing agents should not preclude proteomic analysis by tandem MS/MS in many cases. Although a greater mass of protein extracted by solubilization reagents may be necessary for downstream applications (e.g., gel electrophoresis), or for maximum recovery from degraded, low-protein samples, we strongly recommend that, when conducting proteomic analyses of bone (fossil or extant) the demineralization and wash products be retained and investigated, rather than discarded.

Beyond that overarching point, we also observed some general trends in the extractome recovered by each discrete step of the extraction protocols. Among demineralization fractions, larger volumes of reagent (relative to bone mass) generally resulted in a higher bulk protein yield and greater numbers of peptides, protein diversity, and sequence coverage identified by downstream MS analyses, with some exceptions and caveats. Increasing the relative volume of either HCl or EDTA from 5 mL/g to 20 mL/g led to double or triple the bulk protein yield (Fig. S2), and HCl or EDTA showed similar protein recovery at similar volumes. Notably, a brief incubation of the pellet in NaOH prior to application of EDTA led to a slightly higher protein yield for this reagent (e.g., 20-N/EDTA1-D vs. 20-EDTA-D), but the reason for this is unclear. Although increasing reagent volume increased protein yield, additional incubations in fresh reagent did not generate a substantial amount of additional bulk protein during demineralization (Method 7, 20-N/EDTA1-D vs. 20-N/EDTA2-D). For HCl fractions, dialysis versus precipitation made virtually no difference for total protein yield (Methods 1–4).

MS analyses showed that 20-HCl (Method 4) incubations resulted in the highest degree of diversity in recovered proteins, with precipitated fractions resulting in a degree of recovered protein diversity only slightly higher than dialysis. Furthermore, only the two 20-HCl fractions recovered [alpha-2 antiplasmin], coagulation factor IX, osteonectin (P36377), and osteopontin (Table 2). When compared with other protocols, 20 mL/g HCl resulted in the greatest percentage of peptide coverage for 10 proteins (12K serum protein, [alpha-2-HS-glycoprotein] cystatin, decorin, [dentin matrix protein 1], gremlin-1, histone 2A, lumican, matrix Gla, and osteocalcin) and resulted in ∼20% and ∼30% peptide coverage for collagen I alpha 1 and alpha 2, respectively (Table 3). 5-HCl-P incubations (Methods 1–3) performed similarly to larger-volume HCl fractions in protein diversity (*p* > 0.05) and coverage. 5-HCl-P was the only fraction to recover [heterochromatin associated protein], and resulted in the best peptide coverage for 8 other proteins, including 12K serum protein, [EXFAB], gallinacin-7, histone 2B, lysozyme C, myeloid protein, ribonuclease homolog, and tetranectin. Conversely, 5-HCl-D fractions resulted in similar peptide counts and percent coverage for collagen I alpha 1 and alpha 2 (∼20% and ∼30%, respectively) as other HCl fractions, but a much lower number and percent coverage for identified NCPs (Table 3). Although 20-HCl treatments only performed marginally better than precipitated 5-HCl-P, the larger total protein yield of the 20-HCl fractions (∼4.5 mg/g vs. ∼1 mg/g) lead us to recommend larger volumes for extraction over smaller volumes for modern samples (there are additional concerns for fossil samples; see below). Finally, it should also be noted that osteocalcin, a small, acidic NCP intimately associated with bone mineral (Buckley et al., 2008; Ostrom et al., 2006; Ostrom et al., 2000), was consistently found in HCl fractions, but *only* in HCl fractions (Table 2). This may be the result of osteocalcin decarboxylation in an acidic reagent.

Proteins identified within EDTA fractions were, on average, less diverse than those identified within HCl fractions, but were in some ways complementary to the regions of the proteome retrieved by HCl. We tested 4 different fractions of EDTA: two combined serial incubations of EDTA (6-EDTA-D, Method 5), 20-EDTA-D (Method 6), and two separated serial incubations of 20 mL/g EDTA, prior to which the bone powder had been briefly incubated in 0.1 M NaOH (20-N/EDTA1-D and 20-N/EDTA2-D, Method 7). The 20-N/EDTA-D incubations out-performed both non-NaOH prepped EDTA fractions for total PSMs and peptides identified, overall protein diversity, and sequence coverage. Notably, the second serial incubation was slightly richer than the first, resulting in one additional protein (gremlin-1) and slightly greater or equal peptide coverage than the initial incubations for 12 of the 16 proteins they shared for which coverage was tracked, despite having a substantially lower overall protein yield as measured by BCA (∼6.5 mg/g in 20-N/EDTA1-D vs. ∼0.5 mg/g in 20-N/EDTA2-D, Fig. S2A). Taken together, 20-N/EDTA-D incubations had the second highest degree of identified protein diversity of all reagents tested, after 20-HCl and 5-HCL-P fractions. Although EDTA fractions did not identify any proteins that were not also identified in other treatments, 20-N/EDTA-D fractions resulted in the highest percentage of peptide coverage for multiple key NCPs, including apolipoprotein, serum albumin (P19121), osteonectin (F1P291), [PEDF], secreted phosphoprotein 24, and vitronectin. However, although this method recovered abundant NCPs, 20-N/EDTA-D extractions recovered only marginal amounts of collagen I relative to other treatments, with 7.2–16.2% peptide coverage of alpha 1 and only 7.2–8.8% of alpha 2 (HCl fractions recovered ∼20% and ∼30% peptide coverage, respectively). This suggests that EDTA recovers less collagen than HCl overall, but contains a greater proportion of NCPs per µg of extracted protein than HCl extraction products. To test this hypothesis, we performed ELISA on 1 µg aliquots of extracted protein from demineralization fractions using antibodies raised against purified collagen. The results (Fig. S3A) are consistent with this interpretation; absorbance values, which are a proxy for antibody-antigen interactions (Paulie, Perlmann & Perlmann, 2005), are highest in HCl fractions, and lowest in 20-N/EDTA-D fractions, suggesting that less collagen I is present per µg EDTA-extracted bone proteins, leaving the presence of other NCPs that comprise bone tissue to account for the difference.

Conversely, 20-EDTA-D fractions, not pre-treated with NaOH, did comparatively poorly, resulting in some of the lowest numbers of PSMs, peptides, proteins, and percent coverage of all fractions. Unlike HCl, lower volumes of EDTA without NaOH pretreatment did better than their higher-volume counterpart (15 proteins identified from 6-EDTA-D vs. 9 proteins identified from 20-EDTA-D). This discrepancy could be caused by the difficulty of removing EDTA from solution (e.g., Pereira-Mouries et al., 2002); thus, fractions with larger volumes of reagent may retain more EDTA after dialysis and lyophilization, causing greater interference during ionization and poorer MS results. However, both high and low volumes of EDTA were much less efficient than either of the two serial incubations of 20-N/EDTA-D. The reason for increased recovery in EDTA fractions pretreated with NaOH is unclear, and must be evaluated more in depth. The purpose of this step in previous published protocols has been to remove NCPs from the bone (Liu et al., 2012) and it is possible that, as opposed to completely removing the NCPs prior to demineralization, brief incubation in NaOH made them more accessible for removal by EDTA, given that 20-N/EDTA-D fractions resulted in greater sequence coverage and more peptides of numerous NCPs and less coverage/fewer peptides of collagen I than all other fractions (Table 3). Since NaOH is used to buffer EDTA from an acidic to an alkaline pH (e.g., pH 8), it is possible that slightly more basic conditions are optimal for NCP extraction from bone by EDTA. This hypothesis, however, remains to be fully tested. In any case, our data suggest that a brief incubation in NaOH increases the potential recovery of NCPs with EDTA demineralization, resulting in even greater peptide coverage of some proteins than HCl or any subsequent solubilization step, and should be considered as a possible component of methods where EDTA will be used for demineralization or initial extractions.

Solubilization fractions generally extracted a larger yield of bulk protein (per gram of bone analyzed) than demineralization fractions (Fig. S2), but resulted in a lower degree of diversity in identified proteins from every 20 µg protein sample analyzed by MS (Fig. 2 and Table 2). Furthermore, the proteins that were identified from the solubilization fractions were mostly redundant with those recovered in the previous demineralization step (Fig. 3 and Table S3). There were two solubilization fractions that deviated from this trend: 20-H/SDS-P (Method 2) and 20-H/ABC-SV (Method 4). For SDS, the precipitated fraction displayed the most protein diversity of any solubilization fraction (24 proteins identified), despite the fact that, except for acetic acid, it had the lowest overall protein yield (∼1.5 mg/g). Additionally, 20-H/SDS-P obtained the greatest or equal coverage for four proteins (collagen I alpha 1, hemoglobin beta, histone 4, lumican) and resulted in a greater or approximately equal degree of sequence coverage than HCl fractions for four additional proteins, *after* HCl had already been applied to the bone powder (Table 3). When compared solely to other solubilization fractions, 20-H/SDS-P obtained the greatest sequence coverage of 10 of the 16 proteins that were identified in these fractions for which coverage was tracked. The most significant unique feature of SDS in this study is that it seemed to preferentially extract hemoglobin compared to other fractions. Although various fractions of HCl, Urea, and ABC contained, at most, one peptide of hemoglobin alpha-A *or* beta, precipitated SDS contained two peptides of hemoglobin alpha-A and seven peptides of hemoglobin beta (Table 2). To support comparative efficiency of SDS for hemoglobin extraction, we performed ELISA on protein extracts from different reagents using anti-alligator hemoglobin antibodies (see above). The low, but positive signal from HCl and 20-H/Urea-D extracts are consistent with the isolated hemoglobin peptides found in those extractions (Fig. S3B), and the corresponding spike of MS hemoglobin data in SDS is also seen in ELISA (Fig. S3C). Interestingly, the most absorbance observed in ELISA is from dialyzed SDS, which showed three times more absorbance than precipitated SDS when incubated with the same antibodies under the same conditions. This is peculiar, because although 20-H/SDS-D extracted much more bulk protein than 20-H/SDS-P, the dialyzed fractions did very poorly in MS, generating some of the lowest peptide, protein, and coverage results (Fig. 2B–2C and Table 3). We suspect that, rather than a lack of protein diversity in the sample, the poor MS results of 20-H/SDS-D were caused by interference from remaining SDS detergent during ionization. As an anionic detergent, SDS is difficult to remove through dialysis (Naglak, Hettwer & Wang, 1990), and even trace amounts can cause signal suppression in MS by forming adducts with peptides, increasing sample conductivity, and other factors (Fridriksson, Baird & McLafferty, 1999; Shieh, Lee & Shiea, 2005). Therefore, although dialysis of SDS may retain more bulk protein than precipitation, potentially with greater richness of hemoglobin peptides than any other studied sub-extraction, our data show that precipitation is a comparatively better SDS removal method for downstream MS applications.

20-H/ABC-SV (Method 4) resulted in the best peptide coverage for collagen I alpha 2 (43.7%) and transthyretin. Although it did not rank in the top five treatments for resulting peptide counts or identified protein diversity, when compared solely to other solubilization fractions, it resulted in the best coverage of certain key bone and serum proteins including [alpha-2-HS-glycoprotein], and osteonectin (F1P291). Dialyzed and speed vacuumed 20-H/ABC resulted in variable identifications of low-abundance proteins, but generally produced the same degree of protein diversity (15 proteins identified) and similar peptide counts/sequence coverage for the major constituent proteins (e.g., collagen I, apolipoprotein, serum albumin) (Tables 2 and 3). Thus, the greatest advantages conferred by speed vacuuming ABC over other treatments are: (1) more bulk protein available for downstream applications, and (2) time saved, as it can be performed quickly over the course of hours instead of days. It should be noted that although ABC performed fairly well after demineralization of the bone powder with HCl (Method 4), identical incubations of ABC after demineralization with EDTA (20-E/ABC-SV, Method 7), resulted in the identification of comparatively fewer peptides and lower protein diversity (Tables 2 and 3), suggesting that HCl is a more optimal demineralizing treatment for bone prior to ABC than EDTA.

In general, GuHCl fractions obtained fairly consistent results, regardless of reagent used for pre-demineralization (e.g., HCl, Method 1 or EDTA, Method 5). Among solubilization fractions, whether precipitated or dialyzed, demineralized with HCl or EDTA, 4 M or 6 M concentration, GuHCl extracted less than or equal bulk protein as 20-H/ABC-D and more than SDS fractions (Fig. 2B), while in MS analyses, fewer peptides and proteins were identified in GuHCl fractions than in ABC or precipitated SDS fractions. Within GuHCl fractions, 5-H/GuHCl-D produced the richest MS data, identifying 5+ proteins compared to other GuHCl fractions and obtaining the best (or equal coverage) for 9 of the 10 proteins identified in GuHCl for which coverage was tracked (Table S3). 20-E/A/GuHCl-D obtained additional bulk protein from bone after the pellet had previously been incubated in EDTA and ABC (Method 6), but the specific proteins obtained were nearly all found in previous fractions of the method (Fig. 3F). Thus, while serial incubations of GuHCl after ABC may obtain additional extracted protein for downstream analyses that require large volumes of protein (e.g., gel electrophoresis), it does not appear to expand access to other regions of the bone proteome that were not previously obtained in other fractions.

20-H/Urea-D extracted less bulk protein than all other fractions save SDS, but resulted in fewer peptides and a lower degree of identified protein diversity in MS analyses than SDS, and was roughly on par with 5-H/GuHCl-D in terms of identified protein diversity and peptide coverage. This method identified only one protein (Histone-lysine N-methyltransferase) that was unavailable in other extractions (Table 2). The most notable thing about the Urea extractions is that samples which did not contain protein (i.e., blank buffer controls) generated large amounts of non-protein solids during precipitation, heavily skewing control data (Fig. S1B). We tested this anomaly multiple times, and found that high levels of non-protein material only precipitated when the supernatant was void of protein—spiking bovine serum albumin into the buffer supernatant prevented this reaction upon addition of acetone. This phenomenon precludes precipitation of Urea as a viable buffer removal method for any bone protein study that requires the inclusion of negative controls.

Acetic acid, with and without pepsin, extracted such a low amount of total protein from bone powder that these fractions were not tested in MS analyses (Fig. S2B). Acetic acid has been used as a solubilization agent in protocols designed to extract purified collagen I from skin and bones, to the exclusion of other NCPs, for inoculation to produce collagen I specific antibodies (Liu et al., 2012; Singh et al., 2011). The small amount of overall bone protein recovered from these fractions may be the result of hydrolyzed collagen being lost through the 20K MWCO dialysis membrane along with the smaller NCPs. These results suggest that significant amounts of bone must be processed for a proteomic study that requires multiple assays, and that this method would be particularly ill-suited for studies in which available bone material is limited.

When considered as a whole, these extractome data allow us to make recommendations for how discrete steps in methods may be combined to maximize the breadth and coverage of the bone proteome identified by MS analyses. By combining the two demineralization fractions that recovered the most diverse set of peptides (20-HCl-P and 20-N/EDTA-D) with the solubilization fraction producing a similar diversity (20-H/SDS-P)—only three of the 19 fractions that were analyzed by tandem MS/MS—we can account for 51 of the 55 proteins identified across all fractions, or 92.7% of the total protein diversity identified in the entire study (Fig. 3H). Furthermore, these three fractions include those with the highest (or nearly highest) coverage of all proteins for which coverage data was tracked, thus optimizing this model for degree of identified protein coverage as well as diversity. To combine these sub-extractions into one protocol, Method 2 (Table 1) need only be slightly modified by increasing the initial demineralization volume to bone weight ratio to 20 mL/g, precipitating both fractions as described, and performing a tandem extraction of additional bone material in which the sample is treated with 0.1 M NaOH prior to application of 20 mL/g EDTA.

### Implications for paleoproteomics and future directions

Because the extant chicken bones used for these analyses were not subject to the diagenetic factors that can cause chemical alterations in fossil proteins (e.g., deamidation, carboxymethylation of lysine, loss of hydroxylations to proline (Cleland et al., 2016; Cleland, Schroeter & Schweitzer, 2015; Hill et al., 2015)), it is unclear whether the disparity of efficiencies between protocols observed here would be similar if conducted on a fossil sample. Indeed, given the chemical differences in depositional environments experienced by fossils from different localities, it is possible that similar comparison analyses of 10 different fossil specimens would yield 10 different results. Regardless, the current data conducted on chicken bone may have implications for paleoproteomic studies that can be explored in future fossil analyses.

First, the demineralization fractions (e.g., HCL or EDTA), shown here to have higher proteomic diversity per µg in MS, are commonly discarded in paleoproteomics experiments, presumably because their low-yields are considered negligible (e.g., Buckley & Wadsworth, 2014; Cappellini et al., 2012; Wadsworth & Buckley, 2014). This may be counterproductive in studies targeting NCPs of extinct taxa. Collagen I is the most abundant protein in bone tissue, but it contains minimal informative sequence variation between species; therefore, improving access to NCPs in the bone proteomes of extinct taxa may significantly increase our access to phylogenetically relevant data (Cleland, Voegele & Schweitzer, 2012; Schweitzer, Schroeter & Goshe, 2014; Wadsworth & Buckley, 2014). However, studies targeting NCPs in ancient bone that discard, or simply do not analyze, the demineralization fraction and subsequent water “washes” may be discarding the NCPs they seek. For example, a recent study by Wadsworth & Buckley (2014) investigated proteome degradation in archaeological bone up to one million years old by performing tandem MS/MS on protein extracts solubilized by GuHCl after bone mineral was demineralized in HCl. Our data suggest that additional analyses of the HCl products in similar studies may provide an even wider window into the proteome of these specimens than could be accessed by GuHCl alone, potentially allowing access to more NCPs in older fossils.

Additionally, the observed preferential extraction of hemoglobin from bone by SDS warrants testing this reagent for its possible utility in targeting hemoglobin extraction from fossils. Hemoglobin is a serum protein that can potentially shed light on the evolution of endothermy (Schweitzer & Marshall, 2001) and organismal response to climate change (Campbell et al., 2010), in addition to carrying greater phylogenetic and paleobiological signal than other, more conserved proteins (e.g., Weber, 1995). Although the breakdown products of hemoglobin have been identified in dinosaurs (Schweitzer et al., 1997), peptide sequences from dinosaur fossils have remained elusive. When combined with more advanced techniques for anionic detergent removal from the supernatant (e.g., precipitation kits specifically designed for SDS), SDS may be superior to previous methods for hemoglobin extraction.

However, there are some caveats in applying the results of these experiments on extant bone to paleoproteomic studies. For example, while large-volume fractions of reagents (e.g., 20 mL/g) did better in MS analyses than their lower volume counterparts, the comparative dilution of peptides in these sample volumes may cause greater peptide loss from adsorbance on the sides of pipette tips, sample tubes, dialysis tubing, etc. (Chambers, 2013) which may be a serious problem when studying ancient specimens in which preserved protein is already scarce. The tandem extractions using both HCl and EDTA we recommend for modern bone require processing twice as much bone powder as any single protocol, which may be a concern in studies where sample material is extremely limited. In addition, the total weight of the extraction products of these three reagents (HCl, EDTA, SDS) *combined* was five times less than bulk protein extracted per gram of bone from only one extraction of speed-vacuumed ABC. Thus, although MS only requires micrograms worth of protein, paleoproteomic studies should consider pairing a diversity-rich, low-yield demineralization fraction (e.g., 20-HCl-P) with a higher-yield solubilization fraction (e.g., 20-H/ABC-SV) and analyzing *both* fractions, or processing large amounts of sample material as feasible. In either case, for studies specifically seeking NCPs in low protein samples (e.g., osteonectin in fossils) it is imperative that any demineralization fractions be analyzed rather than discarded, regardless of comparatively minute amounts of extract. Conversely, extraction protocols that do not include a separate demineralization stage (e.g., Cleland & Vashishth, 2015; Hill et al., 2015) may be particularly well suited for the recovery of NCPs from fossils, and should be further explored.

## Conclusions

Based on our data, we make the following conclusions and recommendations for future proteomic studies of bone:

• Dry pellet weight is an inaccurate predictor of protein yield; we recommend against the use of pellet weight as a proxy for protein yield in bone protein studies, whether for assessing efficiency of extractions or preparing subsequent samples for further assays (e.g., ELISA) or proteomic analyses (MS).
• Demineralization fractions of extraction protocols produce low total protein yield, but are richer in protein diversity and coverage per µg. These should be analyzed in MS studies, *not* discarded, particularly in studies that are examining the bone proteome broadly. Whether the sub-extraction was a demineralization step or a solubilization step was the single most important factor affecting diversity and coverage of the NCPs identified in this study.
• As fractions that yielded the most bulk protein overall did not also yield the broadest extractome, bone protein studies should factor this disparity into their experimental design when accounting for their specific target proteins, the sample quantity requirements of their downstream assays, and their desired level of proteome coverage.
• The greatest degree of bone proteome coverage was achieved by combining 3 low-yield fractions in two tandem extractions. While our data show that this combined method works well for modern bone, its application to specimens with a diminished proteome or limited sample material may not be ideal.
• For samples in which analysis of a separate demineralization fraction is not feasible, alternative methods that do not fractionate demineralization, or do not demineralize (e.g., Cleland & Vashishth, 2015; Hill et al., 2015), should be considered to avoid loss of identified protein diversity.

##  Supplemental Information

10.7717/peerj.2603/supp-1 Figure S1 Graphs of pellet weight (light columns) and protein weight recovered as calculated by BCA (dark columns) for (A) demineralization fractions, and (B) solubilization fractions In all instances, the pellet weight produced after final clean-up of the fraction supernatant was more than half the actual weight of protein extracted, suggesting that no method tested was able to completely remove all salts, detergents, and other non-proteinaceous materials from the extraction products. Additionally, pellet size was not predictive of *relative* protein recovery. For example, in (B), although the pellets produced by 8-E/GuHCl fractions (Method 5) were larger than for pellets produced by 20-H/ABC-SV (Method 4), the ABC yielded more overall protein.

10.7717/peerj.2603/supp-2 Figure S2 Total protein yield obtained from (A) demineralization fractions, and (B) solubilization fractions (Please note the differences in scale between figures A and B). (A) Among demineralization fractions, large-volume extractions yielded a greater amount of protein regardless of the type of reagent. The greatest yield was generated by 20-N/EDTA. (B) Among solubilization fractions, 20-ABC-SV fractions had the largest yield, whether preceded by HCl or EDTA. 20-H/ABC-D, and all varieties of GuHCl fractions had the next greatest yield, about half of 20-ABC-SV samples.

10.7717/peerj.2603/supp-3 Figure S3 ELISA absorbance values for demineralization fractions at 270 min against anti-chicken collagen I antibodies (1:1000) The absorbances observed for EDTA fractions were lower than those obtained for HCl fractions, suggesting that per µg of extracted protein, EDTA possessed less collagen I and more NCPs. The nearest absorbance value to those obtained for EDTA was 20-HCL-P, which was also observed to have a greater diversity of NCPs than other fractions (see Table 2). Values are based on averages of duplicate absorbance readings.

10.7717/peerj.2603/supp-4 Figure S4 ELISA absorbance values for (A) demineralization fractions at 180 min against anti-alligator hemoglobin antibodies (1:850) and (B) solubilization fractions at 180 min (1:700) Immunoreactivity to anti-alligator hemoglobin was low, but positive in most demineralization fractions (A), and absent from most solubilization fractions except SDS (B), where a substantial signal was observed in both dialyzed and precipitated SDS samples (20-H/SDS-D and 20-H/SDS-P). 20-H/SDS-D obtained absorbance values 3 times higher that precipitated samples, although hemoglobin was identified in MS analyes of 20-H/SDS-P and *not* in 20-H/SDS-D. Values are based on averages of duplicate absorbance readings.

10.7717/peerj.2603/supp-5 Table S1 False discovery rate (FDR), peptide/protein score, MS and MS/MS scan statistics DB search statistics for all samples, listed by PEAKS search file. Two samples for each reagent were analyzed by tandem MS/MS, then searched together in PEAKS, producing one search node. The FDR filters for each node were set to 1% FDR for peptide spectral matches (PSMs), and 1% FDR for proteins OR a protein score of −log_10_*p* ≥ 20 (whichever was more strict) plus one unique peptide. The corresponding peptide FDR and expect scores, as well as the number of MS and MS/MS scans analyzed, are listed for all searches.

10.7717/peerj.2603/supp-6 Table S2 Protein accession numbers and descriptions Comprehensive list of the 55 proteins recovered across all fractions in this study, including name, accession number, and description. Note that 14 of the 55 accession numbers recovered corresponded to proteins listed as “uncharacterized” in UniProt’s chicken database (rows highlighted in green). We used UniProt’s basic local alignment search tool (BLAST) to determine what these proteins corresponded with in other avians. These correspondences are given as the protein [name] (marked with brackets to differentiate them) and are referred to as such in the text for clarity.

10.7717/peerj.2603/supp-7 Table S3 Redundancy of proteins between solubilization and demineralization fractions in each method For five of the seven methods, 60% or more of the proteins identified in the solubilization fractions had already been identified in the demineralization fractions. For method 6, the two solubilization fractions together (20-E/ABC-SV and 20-E/A/GuHCl-D) were 45.45% redundant with the demineralization fraction.

10.7717/peerj.2603/supp-8 Table S4 T-test values for (A) collagen I ELISA results, (B) hemoglobin ELISA results, (C) protein diversity results of all fractions T-test scores are mirrored across the diagonal black line for ease of reference. Results of *P* < 0.05 are yellow, *P* < 0.01 are green. Sample size (n) is equal to 2. For lists of effect sizes (d), see the second sheet of this document. (A) Red cross divides HCl and EDTA fractions on each axis. (B–C) Red cross divides demineralization and solubilization fractions on each axis.

10.7717/peerj.2603/supp-9 Table S5 Master list of peptides analyzed in this study Peptides listed by fraction and sample # (two were analyzed for each fraction). In this list, peptides corresponding to common lab contaminants (e.g., keratin) have been eliminated, and duplicate matches of one spectrum to multiple protein accession IDs have been reduced to a single occurrence. Peptides of proteins that were only identified by 1 peptide across all fractions have been highlighted in red. These were not used in comparison statistics or analyses.

10.7717/peerj.2603/supp-10 Table S6 Peptides identified in samples from Method 1–3, dialyzed HCl (5 mL/g) (5-HCL-D) 1% FDR

10.7717/peerj.2603/supp-11 Table S7 Peptides identified in samples from Method 1–3, precipitated HCl (5 mL/g) (5-HCL-P) 1% FDR

10.7717/peerj.2603/supp-12 Table S8 Peptides identified in samples from Method 4, dialyzed HCl (20 mL/g) (20-HCl-D) 1% FDR

10.7717/peerj.2603/supp-13 Table S9 Peptides identified in samples from Method 4, precipitated HCl (20 mL/g) (20-HCl-P) 1% FDR

10.7717/peerj.2603/supp-14 Table S10 Peptides identified in samples from Method 5, dialyzed EDTA (6 mL/g) (6-EDTA-D) 1% FDR

10.7717/peerj.2603/supp-15 Table S11 Peptides identified in samples from Method 6, dialyzed EDTA (20 mL/g) (20-EDTA-D) 1% FDR

10.7717/peerj.2603/supp-16 Table S12 Peptides identified in samples from Method 7, dialyzed EDTA 1 (20 mL/g), post-NaOH (First incubation) (20-N/EDTA1-D) 1% FDR

10.7717/peerj.2603/supp-17 Table S13 Peptides identified in samples from Method 7, dialyzed EDTA-2 (20 mL/g), post-NaOH (2nd incubation) (20-N/EDTA2-D) 1% FDR

10.7717/peerj.2603/supp-18 Table S14 Peptides identified in samples from Method 1, dialyzed GuHCl (5 mL/g), post-HCl (5-H/GuHCl-D) 1% FDR

10.7717/peerj.2603/supp-19 Table S15 Peptides identified in samples from Method 1, precipitated GuHCl (5 mL/g), post-HCl (5-H/GuHCl-P) 1% FDR

10.7717/peerj.2603/supp-20 Table S16 Peptides identified in samples from Method 2, dialyzed SDS (20 mL/g), post-HCl (20-H/SDS-D) 1% FDR

10.7717/peerj.2603/supp-21 Table S17 Peptides identified in samples from Method 2, precipitated SDS (20 mL/g), post-HCl (20-H/SDS-P) 1% FDR

10.7717/peerj.2603/supp-22 Table S18 Peptides identified in samples from Method 3, dialyzed urea (20 mL/g), post-HCl (20-H/Urea-D) 1% FDR

10.7717/peerj.2603/supp-23 Table S19 Peptides identified in samples from Method 4, dialyzed ABC (20 mL/g), post-HCl (20-H/ABC-D) 1% FDR

10.7717/peerj.2603/supp-24 Table S20 Peptides identified in samples from Method 4, speed vacuum ABC (20 mL/g), post-HCl (20-H/ABC-SV) 1% FDR

10.7717/peerj.2603/supp-25 Table S21 Peptides identified in samples from Method 5, dialyzed GuHCl (8 mL/g), post-EDTA (8-E/GuHCl-D) 1% FDR

10.7717/peerj.2603/supp-26 Table S22 Peptides identified in samples from Method 5, precipitated GuHCl (8 mL/g), post-EDTA (8-E/GuHCl-P) 1% FDR

10.7717/peerj.2603/supp-27 Table S23 Peptides identified in samples from Method 6, speed vacuumed ABC (20 mL/g), post-EDTA (20-E/ABC-SV) 1% FDR

10.7717/peerj.2603/supp-28 Table S24 Peptides identified in samples from Method 6, dialyzed GuHCl (20 mL/g), post-EDTA, post-ABC, (20-E/A/GuHCl-D) 1% FDR

## Additional Information and Declarations

### Data Availability

The following information was supplied regarding data availability: Schroeter E. 2016. Bone proteome extractomics raw data. Figshare: https://dx.doi.org/10.6084/m9.figshare.c.3269222.v1.
