# Supplementary material for: Bone protein “extractomics”: comparing the efficiency of bone protein extractions of Gallus gallus in tandem mass spectrometry, with an eye towards paleoproteomics
Source: PeerJ. 2016 Oct 27;4:e2603. doi: 10.7717/peerj.2603 (PMC5088622; doi:10.7717/peerj.2603)
Supplement: Figure S1 — In all instances, the pellet weight produced after final clean-up of the fraction supernatant was more than half the actual weight of protein extracted, suggesting that no method tested was able to completely remove all salts, detergents, and other non-proteinaceous materials from the extraction products. Additionally, pellet size was not predictive of relative protein recovery. For example, in (B), although the pellets produced by 8-E/GuHCl fractions (Method 5) were larger than for pellets produced by 20-H/ABC-SV (Method 4), the ABC yielded more overall protein. [file peerj-04-2603-s001.pdf]

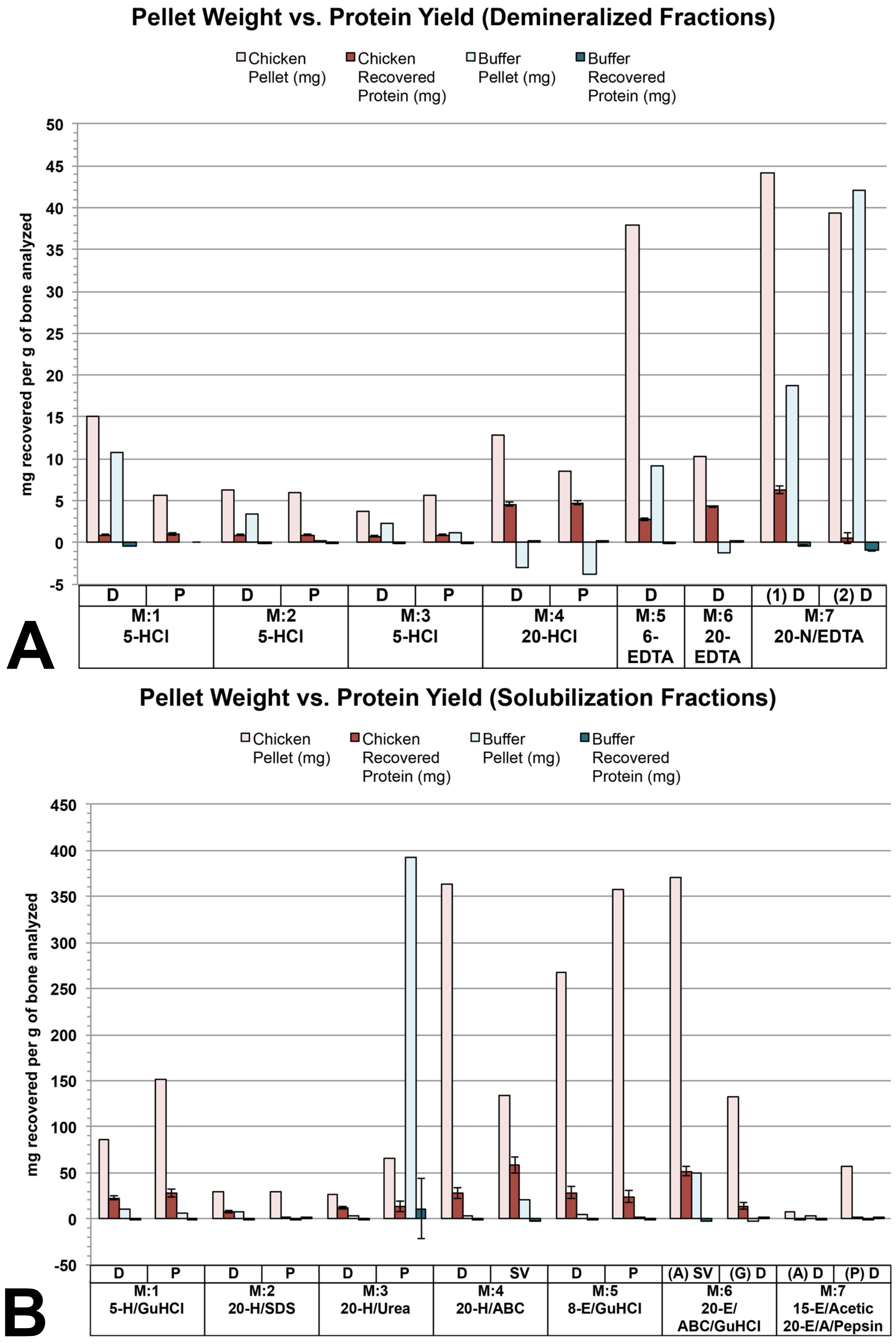
Pellet Weight vs. Protein Yield (Demineralized Fractions)
Chicken Pellet (mg)
Chicken Recovered Protein (mg)
Buffer Pellet (mg)
Buffer Recovered Protein (mg)
mg recovered per g of bone analyzed
50
45
40
35
30
25
20
15
10
5
0
-5
D P D P D P D P D D (1) D (2) D
M:1 5-HCl
M:2 5-HCl
M:3 5-HCl
M:4 20-HCl
M:5 6-EDTA
M:6 20-EDTA
M:7 20-N/EDTA
A
Pellet Weight vs. Protein Yield (Solubilization Fractions)
Chicken Pellet (mg)
Chicken Recovered Protein (mg)
Buffer Pellet (mg)
Buffer Recovered Protein (mg)
mg recovered per g of bone analyzed
450
400
350
300
250
200
150
100
50
0
-50
D P D P D P D SV D P (A) SV (G) D (A) D (P) D
M:1 5-H/GuHCl
M:2 20-H/SDS
M:3 20-H/Urea
M:4 20-H/ABC
M:5 8-E/GuHCl
M:6 20-E/ ABC/GuHCl
M:7 15-E/Acetic 20-E/A/Pepsin
B
