# Supplementary material for: Bone protein “extractomics”: comparing the efficiency of bone protein extractions of Gallus gallus in tandem mass spectrometry, with an eye towards paleoproteomics
Source: PeerJ. 2016 Oct 27;4:e2603. doi: 10.7717/peerj.2603 (PMC5088622; doi:10.7717/peerj.2603)
Supplement: Figure S2 — (Please note the differences in scale between figures A and B). (A) Among demineralization fractions, large-volume extractions yielded a greater amount of protein regardless of the type of reagent. The greatest yield was generated by 20-N/EDTA. (B) Among solubilization fractions, 20-ABC-SV fractions had the largest yield, whether preceded by HCl or EDTA. 20-H/ABC-D, and all varieties of GuHCl fractions had the next greatest yield, about half of 20-ABC-SV samples. [file peerj-04-2603-s002.pdf]

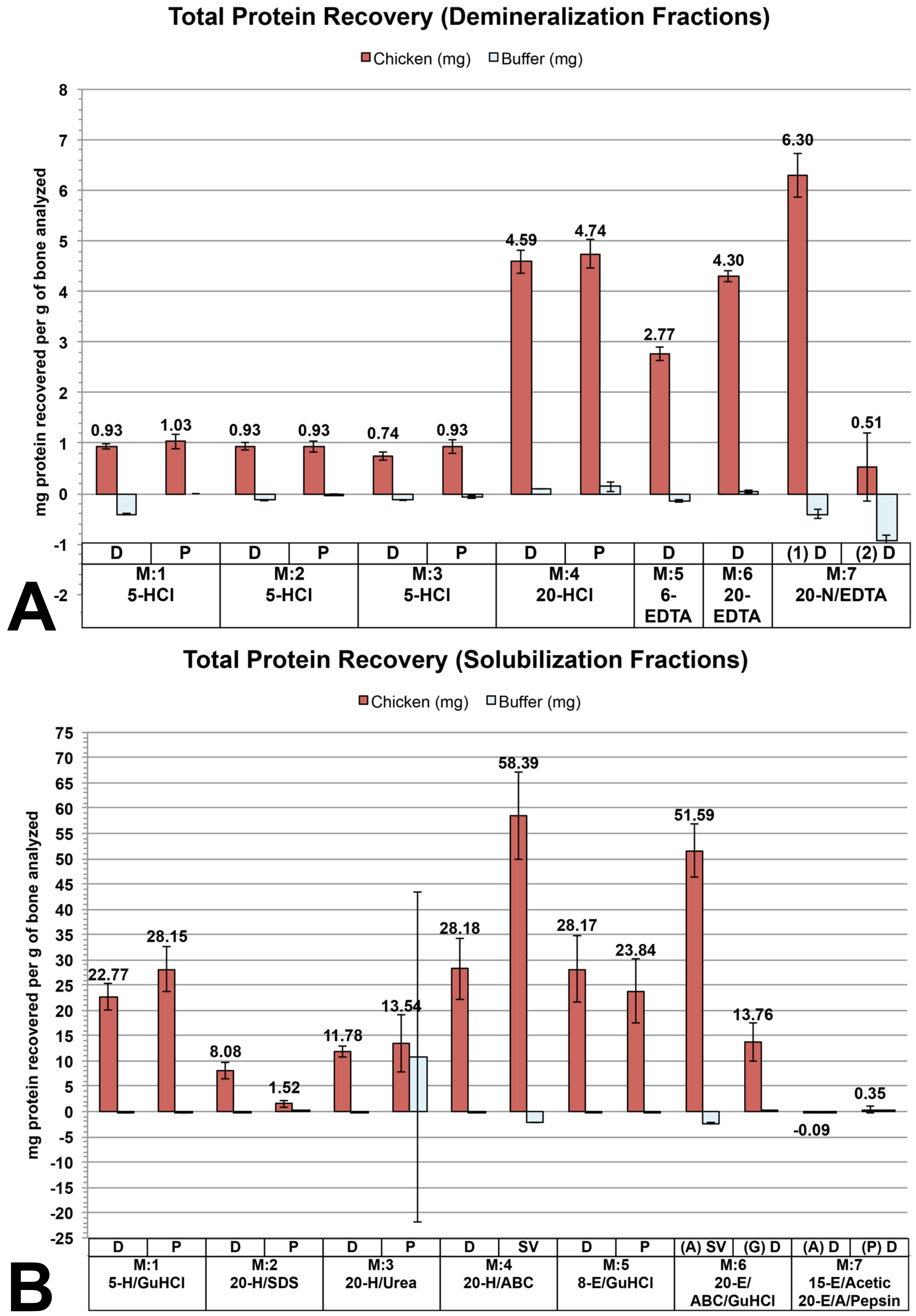

Total Protein Recovery (Demineralization Fractions)
Chicken (mg)
Buffer (mg)
mg protein recovered per g of bone analyzed
8
7
6
5
4
3
2
1
0
-1
-2
0.93
1.03
0.93
0.93
0.74
0.93
4.59
4.74
2.77
4.30
6.30
0.51
D
P
D
P
D
P
D
P
D
D
(1) D
(2) D
M:1
5-HCl
M:2
5-HCl
M:3
5-HCl
M:4
20-HCl
M:5
6-
EDTA
M:6
20-
EDTA
M:7
20-N/EDTA
A
Total Protein Recovery (Solubilization Fractions)
Chicken (mg)
Buffer (mg)
mg protein recovered per g of bone analyzed
75
70
65
60
55
50
45
40
35
30
25
20
15
10
5
0
-5
-10
-15
-20
-25
22.77
28.15
8.08
1.52
11.78
13.54
28.18
58.39
28.17
23.84
51.59
13.76
-0.09
0.35
D
P
D
P
D
P
D
SV
D
P
(A) SV
(G) D
(A) D
(P) D
M:1
5-H/GuHCl
M:2
20-H/SDS
M:3
20-H/Urea
M:4
20-H/ABC
M:5
8-E/GuHCl
M:6
20-E/
ABC/GuHCl
M:7
15-E/Acetic
20-E/A/Pepsin
B
