# Supplementary material for: Bone protein “extractomics”: comparing the efficiency of bone protein extractions of Gallus gallus in tandem mass spectrometry, with an eye towards paleoproteomics
Source: PeerJ. 2016 Oct 27;4:e2603. doi: 10.7717/peerj.2603 (PMC5088622; doi:10.7717/peerj.2603)
Supplement: Figure S3 — The absorbances observed for EDTA fractions were lower than those obtained for HCl fractions, suggesting that per µg of extracted protein, EDTA possessed less collagen I and more NCPs. The nearest absorbance value to those obtained for EDTA was 20-HCL-P, which was also observed to have a greater diversity of NCPs than other fractions (see Table 2). Values are based on averages of duplicate absorbance readings. [file peerj-04-2603-s003.pdf]

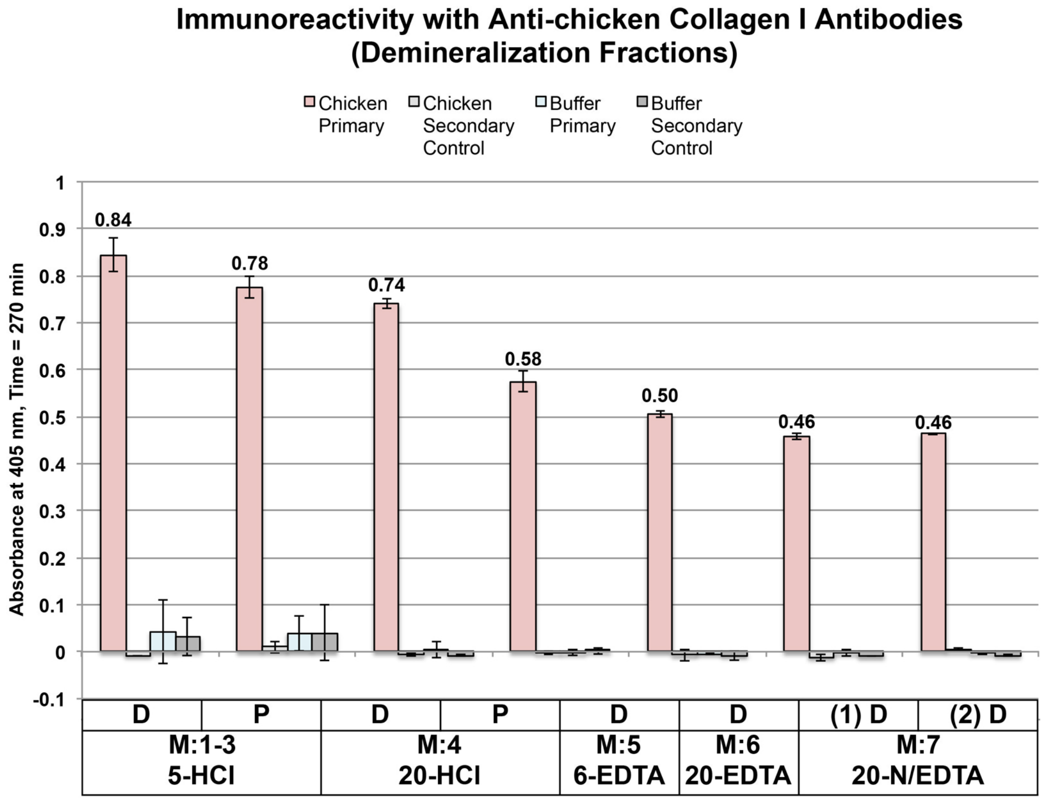
Immunoreactivity with Anti-chicken Collagen I Antibodies
(Demineralization Fractions)
Chicken Primary
Chicken Secondary Control
Buffer Primary
Buffer Secondary Control
Absorbance at 405 nm, Time = 270 min
1
0.9
0.8
0.7
0.6
0.5
0.4
0.3
0.2
0.1
0
-0.1
0.84
0.78
0.74
0.58
0.50
0.46
0.46
D
P
D
P
D
D
(1) D
(2) D
M:1-3
5-HCl
M:4
20-HCl
M:5
6-EDTA
M:6
20-EDTA
M:7
20-N/EDTA
