# Supplementary material for: Bone protein “extractomics”: comparing the efficiency of bone protein extractions of Gallus gallus in tandem mass spectrometry, with an eye towards paleoproteomics
Source: PeerJ. 2016 Oct 27;4:e2603. doi: 10.7717/peerj.2603 (PMC5088622; doi:10.7717/peerj.2603)
Supplement: Figure S4 — Immunoreactivity to anti-alligator hemoglobin was low, but positive in most demineralization fractions (A), and absent from most solubilization fractions except SDS (B), where a substantial signal was observed in both dialyzed and precipitated SDS samples (20-H/SDS-D and 20-H/SDS-P). 20-H/SDS-D obtained absorbance values 3 times higher that precipitated samples, although hemoglobin was identified in MS analyes of 20-H/SDS-P and not in 20-H/SDS-D. Values are based on averages of duplicate absorbance readings. [file peerj-04-2603-s004.pdf]

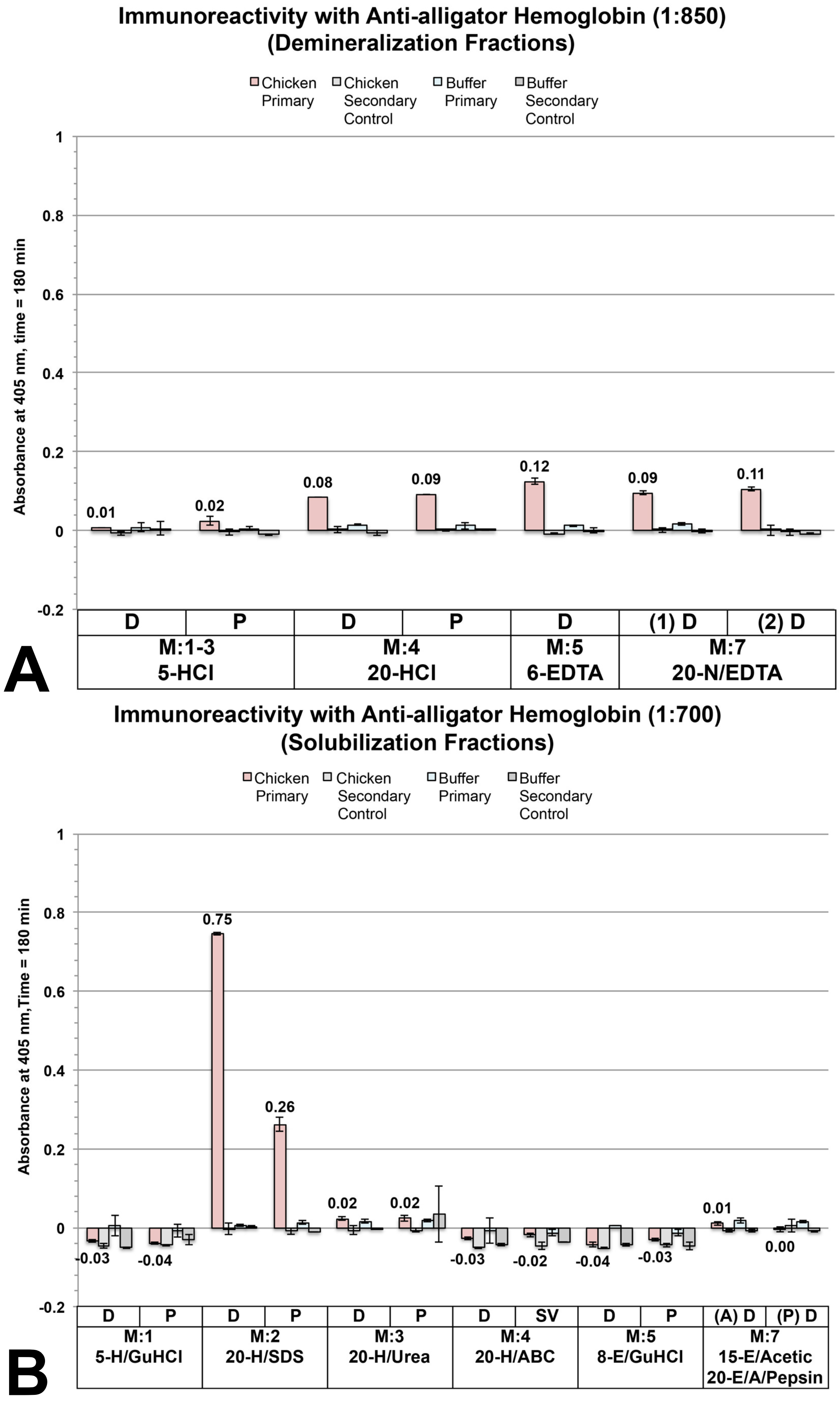

Immunoreactivity with Anti-alligator Hemoglobin (1:850)
(Demineralization Fractions)
Chicken Primary
Chicken Secondary Control
Buffer Primary
Buffer Secondary Control
Absorbance at 405 nm, time = 180 min
1
0.8
0.6
0.4
0.2
0
-0.2
0.01
0.02
0.08
0.09
0.12
0.09
0.11
D
P
D
P
D
(1) D
(2) D
M:1-3
5-HCl
M:4
20-HCl
M:5
6-EDTA
M:7
20-N/EDTA
A
Immunoreactivity with Anti-alligator Hemoglobin (1:700)
(Solubilization Fractions)
Chicken Primary
Chicken Secondary Control
Buffer Primary
Buffer Secondary Control
Absorbance at 405 nm,Time = 180 min
1
0.8
0.6
0.4
0.2
0
-0.2
0.75
0.26
0.02
0.02
0.01
-0.03
-0.04
-0.03
-0.02
-0.04
-0.03
0.00
D
P
D
P
D
P
D
SV
D
P
(A) D
(P) D
M:1
5-H/GuHCl
M:2
20-H/SDS
M:3
20-H/Urea
M:4
20-H/ABC
M:5
8-E/GuHCl
M:7
15-E/Acetic
20-E/A/Pepsin
B
